# Inhibition of endothelial-to-mesenchymal transition in a large animal preclinical arteriovenous fistula model leads to improved remodelling and reduced stenosis

**DOI:** 10.1093/cvr/cvae157

**Published:** 2024-07-26

**Authors:** Yang Xu, Adam Korayem, Ana S Cruz-Solbes, Nirupama Chandel, Tomoki Sakata, Renata Mazurek, Spyros A Mavropoulos, Taro Kariya, Tadao Aikawa, Kelly P Yamada, Valentina D'Escamard, Bhargavi V'Gangula, Andrew H Baker, Lijiang Ma, Johan L M Björkegren, Valentin Fuster, Manfred Boehm, Kenneth M Fish, Rami Tadros, Kiyotake Ishikawa, Jason C Kovacic

**Affiliations:** Cardiovascular Research Institute, Icahn School of Medicine at Mount Sinai, One Gustave L. Levy Place, Box 1014, New York, NY 10029, USA; Division of Vascular Surgery, Department of Surgery, Mount Sinai Hospital, Icahn School of Medicine at Mount Sinai, New York, NY 10029, USA; Cardiovascular Research Institute, Icahn School of Medicine at Mount Sinai, One Gustave L. Levy Place, Box 1014, New York, NY 10029, USA; Cardiovascular Research Institute, Icahn School of Medicine at Mount Sinai, One Gustave L. Levy Place, Box 1014, New York, NY 10029, USA; Cardiovascular Research Institute, Icahn School of Medicine at Mount Sinai, One Gustave L. Levy Place, Box 1014, New York, NY 10029, USA; Cardiovascular Research Institute, Icahn School of Medicine at Mount Sinai, One Gustave L. Levy Place, Box 1014, New York, NY 10029, USA; Cardiovascular Research Institute, Icahn School of Medicine at Mount Sinai, One Gustave L. Levy Place, Box 1014, New York, NY 10029, USA; Cardiovascular Research Institute, Icahn School of Medicine at Mount Sinai, One Gustave L. Levy Place, Box 1014, New York, NY 10029, USA; Cardiovascular Research Institute, Icahn School of Medicine at Mount Sinai, One Gustave L. Levy Place, Box 1014, New York, NY 10029, USA; Cardiovascular Research Institute, Icahn School of Medicine at Mount Sinai, One Gustave L. Levy Place, Box 1014, New York, NY 10029, USA; Cardiovascular Research Institute, Icahn School of Medicine at Mount Sinai, One Gustave L. Levy Place, Box 1014, New York, NY 10029, USA; Cardiovascular Research Institute, Icahn School of Medicine at Mount Sinai, One Gustave L. Levy Place, Box 1014, New York, NY 10029, USA; Centre for Cardiovascular Science, University of Edinburgh, Edinburgh, UK; Department of Pathology, CARIM, Universiteitssingel 50, Maastricht, The Netherlands; Cardiovascular Research Institute, Icahn School of Medicine at Mount Sinai, One Gustave L. Levy Place, Box 1014, New York, NY 10029, USA; Cardiovascular Research Institute, Icahn School of Medicine at Mount Sinai, One Gustave L. Levy Place, Box 1014, New York, NY 10029, USA; Department of Medicine at Huddinge, Karolinska Institutet, Karolinska Universitetssjukhuset, Stockholm, Sweden; Department of Genetics and Genomic Sciences, Institute of Genomics and Multiscale Biology, Icahn School of Medicine at Mount Sinai, New York, NY, 10029, USA; Clinical Gene Networks AB, Stockholm, Sweden; Cardiovascular Research Institute, Icahn School of Medicine at Mount Sinai, One Gustave L. Levy Place, Box 1014, New York, NY 10029, USA; Centro Nacional de Investigaciones Cardiovasculares Carlos III (CNIC), Madrid, Spain; Laboratory of Cardiovascular Regenerative Medicine, Translational Vascular Medicine Branch, National Heart Lung and Blood Institute, NIH, Bethesda, MD, USA; Cardiovascular Research Institute, Icahn School of Medicine at Mount Sinai, One Gustave L. Levy Place, Box 1014, New York, NY 10029, USA; Division of Vascular Surgery, Department of Surgery, Mount Sinai Hospital, Icahn School of Medicine at Mount Sinai, New York, NY 10029, USA; Cardiovascular Research Institute, Icahn School of Medicine at Mount Sinai, One Gustave L. Levy Place, Box 1014, New York, NY 10029, USA; Cardiovascular Research Institute, Icahn School of Medicine at Mount Sinai, One Gustave L. Levy Place, Box 1014, New York, NY 10029, USA; Victor Chang Cardiac Research Institute, Lowy Packer Building, 405 Liverpool St, Darlinghurst 2010, Australia; St. Vincent’s Clinical School, University of NSW, Victoria St, Darlinghurst 2010, Australia

**Keywords:** Endothelial-to-mesenchymal transition, Neointima, Stenosis, Vein graft, Arteriovenous fistula

## Abstract

**Aims:**

Vein grafts are used for many indications, including bypass graft surgery and arteriovenous fistula (AVF) formation. However, patency following vein grafting or AVF formation is suboptimal for various reasons, including thrombosis, neointimal hyperplasia, and adverse remodelling. Recently, endothelial-to-mesenchymal transition (EndMT) was found to contribute to neointimal hyperplasia in mouse vein grafts. We aimed to evaluate the clinical potential of inhibiting EndMT and developed the first dedicated preclinical model to study the efficacy of local EndMT inhibition immediately prior to AVF creation.

**Methods and results:**

We first undertook pilot studies to optimize the creation of a femoral AVF in pigs and verify that EndMT contributes to neointimal formation. We then developed a method to achieve local *in vivo SMAD3* knockdown by dwelling a lentiviral construct containing *SMAD3* shRNA in the femoral vein prior to AVF creation. Next, in Phase 1, six pigs were randomized to *SMAD3* knockdown or control lentivirus to evaluate the effectiveness of *SMAD3* knockdown and EndMT inhibition 8 days after AVF creation. In Phase 2, 16 pigs were randomized to *SMAD3* knockdown or control lentivirus and were evaluated to assess longer-term effects on AVF diameter, patency, and related measures at 30 days after AVF creation. In Phase 1, compared with controls, *SMAD3* knockdown achieved a 75% reduction in the proportion of CD31^+^ endothelial cells co-expressing SMAD3 (*P* < 0.001) and also a significant reduction in the extent of EndMT (*P* < 0.05). In Phase 2, compared with controls, *SMAD3* knockdown was associated with an increase in the minimum diameter of the venous limb of the AVF (1.56 ± 1.66 vs. 4.26 ± 1.71 mm, *P* < 0.01) and a reduced degree of stenosis (*P* < 0.01). Consistent with this, neointimal thickness was reduced in the *SMAD3* knockdown group (0.88 ± 0.51 vs. 0.45 ± 0.19 mm, *P* < 0.05). Furthermore, endothelial integrity (the proportion of luminal cells expressing endothelial markers) was improved in the *SMAD3* knockdown group (*P* < 0.05).

**Conclusion:**

EndMT inhibition in a preclinical AVF model by local *SMAD3* knockdown using gene therapy led to reduced neointimal hyperplasia, increased endothelialization, and a reduction in the degree of AVF stenosis. This provides important proof of concept to pursue this approach as a clinical strategy to improve the patency of AVFs and other vein grafts.

## Graphical Abstract

**Graphical Abstract:**
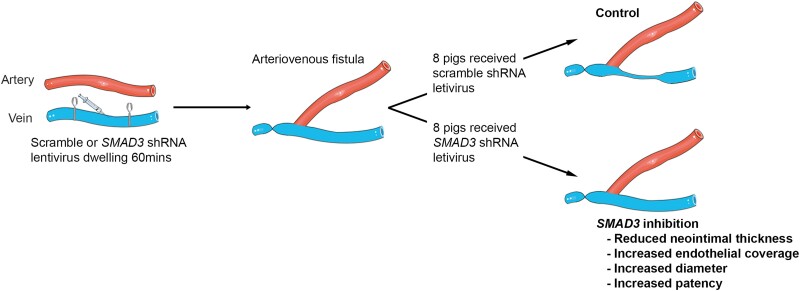
Inhibition of endothelial-to-mesenchymal transition in a large animal arteriovenous fistula (AVF) model mimicking a human haemodialysis fistula by *SMAD3* knockdown with lentiviral gene therapy led to reduced neointimal hyperplasia, increased endothelialization, increased AVF diameter, and potentially to improved AVF patency.

**Time of primary review: 29 days**

**See the editorial comment for this article ‘Whenbigger is better: utilizing large animal models in vein graft surgery to gain insights into endothelial-to-mesenchymal transition’, by C. Becher *et al*., https://doi.org/10.1093/cvr/cvae204.**

## 1. Introduction

Despite significant advances in medical and surgical therapies, changes in global disease prevalence and population demographics are driving an epidemic of cardiometabolic disease.^1,2^ As a treatment that has been practiced for decades, various forms of vein graft surgery, including interposition vein grafting into the arterial circulation, remain mainstay surgical options for the treatment of occlusive atherosclerotic disease.^3^ However, the prognosis of grafted veins is far from ideal. It has been reported that at 12–18 months after surgery, up to 42% of vein grafts experience some level of failure and up to 25% of vein grafts may fail completely.^4,5^ In addition, for saphenous veins that are used for coronary artery bypass graft surgery that does not involve the left anterior descending coronary artery, up to 50% of these grafts are reported to be occluded within 10 years.^5,6^

As a closely related issue, the incidence of end-stage kidney disease is also increasing, and almost half a million Americans were undergoing haemodialysis across 2018–20.^7^ In order to undergo haemodialysis, the creation of an arteriovenous fistula (AVF) is often required. Like interposition vein grafting, the creation of an AVF exposes a segment of the vein to arterial shear forces and pressures. Consistent with this, AVFs also have a high failure rate during the ‘maturation’ period after creation and also during ongoing use for haemodialysis. Specifically, during the first 1–2 months after creation, it has been reported that about 30–50% AVFs fail to mature to an optimally usable state,^8–10^ while for matured AVFs that are used for haemodialysis, the additional failure rate is around 30–40% at 6–12 months.^10,11^

Vein graft and AVF failure arise due to a complicated series of pathobiological processes, primarily thrombosis, adverse vein graft remodelling, and neointimal hyperplasia.^12,13^ In studies dating back over a decade, it was shown that endothelial-to-mesenchymal transition (EndMT) makes a major contribution to neointimal hyperplasia in veins as they remodel and adapt to the arterial environment.^14^ EndMT is a highly dynamic process that involves numerous cellular and subcellular changes and is characterized by endothelial cells losing their usual phenotypic features such as a cobblestone morphology and the expression of typical markers like CD31 and vascular endothelial-cadherin (VE-Cad), as well as the concurrent acquisition of a mesenchymal-like phenotype. Importantly, EndMT is also implicated in endothelial dysfunction and a range of cardiovascular diseases, including atherosclerosis.^15–18^

Over recent years, we have gained significant insights into the mechanisms underlying EndMT. While many pathways are potentially involved, transforming growth factor beta (TGF-β) signalling is known to exert a central governing role over the EndMT process.^19^ Of important relevance, in a mouse vein graft model, the inhibition of TGF-β signalling by *Smad3* knockdown led to a reduction in both EndMT and neointimal hyperplasia, with a significant improvement of vein graft patency.^14^ While subsequent murine studies have confirmed the importance of TGF-β signalling in vein graft remodelling,^19^ until now, there have been no dedicated large animal studies undertaken for the specific purpose of studying EndMT as a clinical therapeutic option.^15,16,20,21^

To address these issues, we created the first dedicated, preclinical, large animal model to specifically study the efficacy of EndMT inhibition. This involved the creation of a femoral AVF in pigs mimicking a haemodialysis fistula, with careful initial evaluation to verify a significant contribution of EndMT to the neointima during vein remodelling. Following this, we proceeded to develop a clinically efficient system for local *in vivo SMAD3* knockdown in the AVF using lentiviral gene therapy. This was a key design aspect of this study, because to have viability as a human therapy local delivery is essential, which also then allows the use of advanced therapies. In this regard, unlike adenoviruses and adeno-associated viruses (AAVs) that are commonly used in therapeutic viral approaches,^22^ lentiviruses are highly effective for transfecting endothelial cells, hence justifying their use in this study.^23^ Finally, we evaluated the efficacy of EndMT inhibition via *SMAD3* knockdown in our femoral AVF model, which was associated with a significant reduction in neointimal hyperplasia, increased endothelialization, and a reduction in the degree of AVF stenosis at 30 days after AVF creation.

## 2. Methods

Additional methods are provided in the Supplementary material including details relating to the lentivirus and its evaluation *in vitro* and *ex vivo* and also the details and surgical procedure for AVF creation.

### 2.1 Pig use, housing, and tissue harvesting

Pigs were housed in the animal facility at the Icahn School of Medicine at Mount Sinai and were fed a standard diet (# 5081, LabDiet Inc., St. Louis, MO, USA) with water being freely available. Animal experiments were approved by the Institutional Animal Care and Use Committee of the Icahn School of Medicine at Mount Sinai, protocol IACUC-2015-0084. All procedures conformed to the NIH Guide for the Care and Use of Laboratory Animals.

On the day of AVF creation, initial anaesthesia was administered as a single intramuscular dose of Telazol® (tiletamine and zolazepam for injection) 8 mg/kg, as well as a dose of pre-operative analgesia with buprenorphine (0.02 mg/kg) intramuscularly. When immobilized, the animal was weighed, placed in a supine position, and then intubated and ventilated with 100% oxygen. General anaesthesia was maintained with 1.5–3.0% isoflurane inhalation throughout the procedure. Details of the surgical creation of the AVF are provided in the Supplementary material.

Subsequent to AVF creation, all animals received aspirin 81 mg daily and clopidogrel 75 mg daily. Post-operative analgesia was provided by administering buprenorphine (0.02 mg/kg) intramuscularly twice daily for 3 days. Intramuscular cefazolin 25 mg/kg was also given twice daily for 10 days after the procedure.

**Figure 1:**
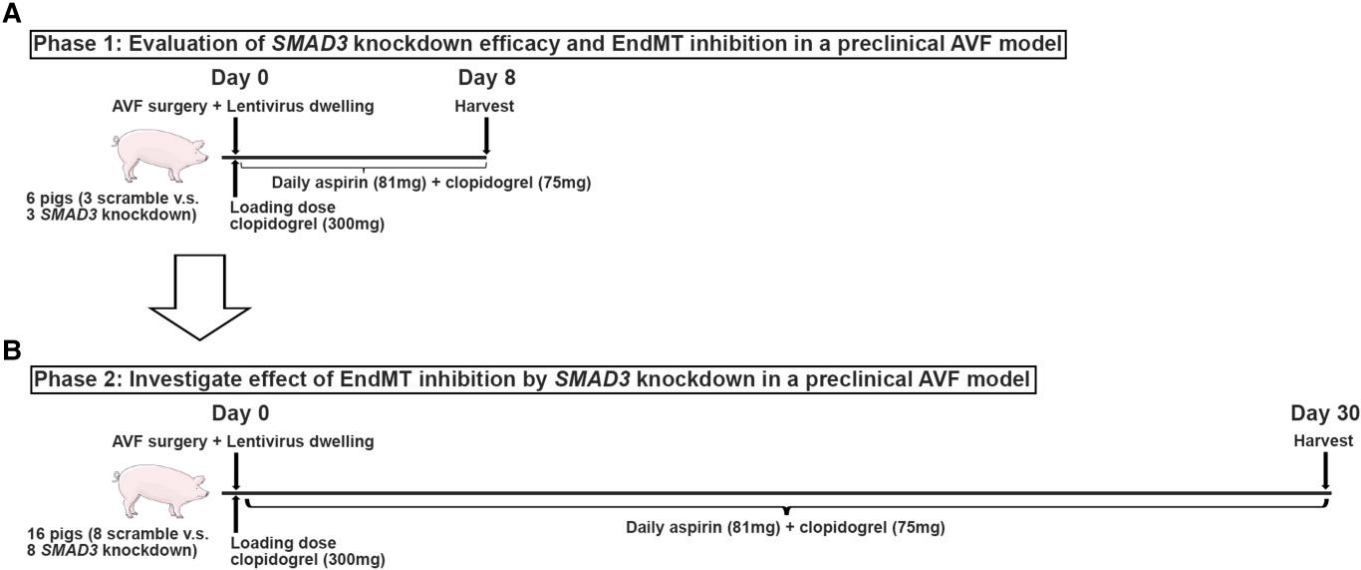
Schematic overview of approach to assessing the efficacy of EndMT inhibition in a preclinical large animal AVF model. (*A*) Phase 1: Evaluation of *SMAD3* knockdown efficacy and EndMT inhibition. The AVF in the right leg was harvested from six pigs, 8 days after AVF creation, where three pigs were randomized to receive lentivirus carrying scramble shRNA and the other three pigs received lentivirus carrying *SMAD3* shRNA. (*B*) Phase 2: Evaluation of the effect and efficacy of EndMT inhibition by *SMAD3* knockdown in this pig AVF model. The AVF in the right leg was harvested from 16 pigs, 30 days after AVF creation, where eight pigs were randomized to receive lentivirus carrying scramble shRNA and the other eight pigs received lentivirus carrying *SMAD3* shRNA.

Pigs were euthanized and tissues were harvested at 15, 8, or 30 days after AVF creation for Phase 0, Phase 1 and Phase 2, respectively (*Figure 1* and Supplementary material online, *Figure S2*). To allow for public holidays and other procedures, the days of harvesting post AVF creation were ±1 day. A single pig was harvested 24 h after AVF creation (see Supplementary material online, *Figure S3C*). Euthanasia was performed while pigs were already under general anaesthesia; isoflurane was increased to 5% for 5 min, and then 50 mg/kg potassium acetate was administered intravenously. Death was confirmed by electrocardiogram.

### 2.2 Ultrasound of AVF

In Phase 2, on the day of planned euthanasia and tissue harvest, animals were placed under general anaesthesia and ultrasound was performed in the supine position. The femoral artery and vein were examined with B mode to evaluate diameter and compressibility. The direction of blood flow was assessed with colour Doppler. Due to signal interference from scar tissue and also because the arterial limb was typically overlying the venous limb, it was not possible to reliably assess the diameter of the venous limb of the AVF by ultrasound.

### 2.3 Angiographic assessment and AVF harvesting

After the ultrasound in Phase 2, and also in Phase 0 and Phase 1, vascular access was obtained in the right carotid artery and external jugular vein using an ultrasound-guided Seldinger technique, followed by the administration of 5000 U unfractionated heparin intravenously. A 5 Fr Judkins right or hockey stick catheter was advanced to the right external iliac artery under fluoroscopic guidance. Contrast agent (ISOVUE®-370, Bracco Diagnostics, Monroe Township, NJ, USA) was injected to visualize the AVF from multiple directions, with the beam projection angle optimized to achieve separation between arterial and venous limbs of the AVF.

After angiography and following humane euthanasia under general anaesthesia, AVF sites were isolated and trimmed of fat and other superfluous tissues, and samples were embedded in both paraffin and optimal cutting temperature compound (OCT). For the Phase 0 studies (see Supplementary material online, *Figure S2*), the left femoral vein was also harvested and embedded in paraffin and OCT.

### 2.4 Histopathology staining

An initial evaluation of sections of the venous limb of the AVF was made to identify differing diameter regions, including the narrowest portion. We then prioritized histopathology (and immunostaining) of differing regions of the venous limb of the AVF, with the narrowest portion used to quantify inner perimeter and calculated lumen area by histopathology (see *Figures 4* and *5* for full details). Paraffin-embedded sections were stained Masson’s trichrome (HT15, Sigma, St. Louis, MO, USA) or elastic van Gieson (EVG) stain (ab150667, Abcam, Waltham, MA, USA) according to the manufacturer’s instructions. Images were acquired using a DMi8 microscope (Leica Microsystems Inc., Deerfield, IL, USA). Fiji software (version 2.0.0-rc-68/1.52w for Mac) was used for image analysis and quantification of inner lumen perimeter, calculated lumen area, vessel wall area, collagen occupied area, and neointimal thickness. Lumen area was calculated from the inner lumen perimeter (i.e. inner lumen circumference) assuming the vessel was circular in cross-section. For lumen area, because we aimed to measure the narrowest portion of the venous limb of the AVF, only one section was used for this measurement. In addition, measurements of the vessel wall area were performed on Masson’s trichrome-stained slides, but we also used EVG-stained sections for guidance to identify the outer boundary of the vessel wall. For the measurement of neointimal thickness, three differing sites were recorded and averaged from a single section.

### 2.5 Immunostaining

For immunofluorescence staining including the *ex vivo* lentiviral article dwelling experiments, frozen sections were initially thawed at room temperature and then fixed by incubating with 4% paraformaldehyde for 10 min. Slides were then washed twice in phosphate-buffered saline (PBS) and permeabilized by applying 0.3% Triton X-100 for 5 min, followed by blocking in 5% bovine serum albumin for 60 min at room temperature. Next, samples were incubated overnight at 4°C with primary antibodies in antibody diluent (S080983-2, DAKO, Carpinteria, CA, USA) at the specified concentrations (see Supplementary material online, *Table S2*). Sections were then washed three times in PBS and incubated for 1 h at room temperature with Invitrogen Alexa Fluor™ 546 and/or Alexa Fluor™ 633 secondary antibodies (see Supplementary material online, *Table S3*). As a single exception, for anti-GFP staining, we used Alexa Fluor™ 488. Finally, slides were washed three times with PBS and mounted with mounting medium containing DAPI (H-1200, Vector Laboratories, Newark, CA, USA). Images were acquired using a confocal microscope (LSM780, Zeiss, White Plains, NY, USA). Data were averaged from at least two to three separate locations per animal. Zen software from Zeiss was used for image analysis.

### 2.6 TUNEL assay

OCT sections were stained with a TUNEL Assay kit (#12156792910, Roche, Pleasanton, CA, USA) according to the manufacturer’s instructions. Images were acquired using a DMi8 microscope (Leica). Data were averaged from at least two to three separate sections per animal.

### 2.7 Statistics

Power calculations for Phase 2 were based on our prior murine studies of EndMT in a vein graft model^14^ and a prior AVF study in pigs.^25^ Assuming a 30% difference in neointimal area between the control and *SMAD3* knockdown groups (we saw a 60% reduction in neointimal area with *Smad3* shRNA in mice^14^), then based on pig femoral AVF dimensions,^25^ seven pigs were required per group to have 80% power to detect a difference at the *P* < 0.05 level. Note that we do not report cross-sectional neointimal area, because due to the extensive vascular remodelling that arose in this AVF model, it was not possible to confidently identify the inner elastic lamina that demarcates the intima-media boundary from the entire cross-section of all samples. Rather, we report mean neointimal thickness from regions where the intima-media boundary could be confidently identified (*Figure 4B*).

All analyses and quantifications were done in a blinded fashion, and no outliers or data points were excluded. For *in vitro* experiments (see Supplementary material online, *Figure S3A*), an unpaired Student’s *t*-test was used. For *in vivo* experiments, the normality of distribution was first tested by Shapiro–Wilk test. For normal distributed data, an *F*-test was used to test for equal variance. If data were equal in variance, an unpaired Student’s *t*-test was used. If data were not equal in variance, an unpaired Student’s *t*-test with Welch’s correction was used. For data that were not normally distributed, a Mann–Whitney *U* test was used. For comparisons in Phase 0 comparing the venous limb of the AVF with the contralateral femoral vein, a paired Student’s *t*-test was used. For categorical data, Fisher’s exact test was used. Statistical analyses were performed using Prism 9 for macOS, and a two-sided *P* value < 0.05 was considered significant. All data are presented as mean ± SD.

## 3. Results

### 3.1 Overview of approach to assess the preclinical efficacy of EndMT inhibition in a large animal model

We first developed a preclinical large animal model of EndMT by optimizing a surgically created right femoral AVF in pigs, i.e. a haemodialysis fistula (see Supplementary material online, *Figures S1* and *S2*). After creating this model (Phase 0), our pre-specified approach to investigate the efficacy of EndMT inhibition in pig AVFs comprised two phases. Phase 1 involved establishing an effective gene delivery system to enable local *in vivo SMAD3* knockdown in this AVF model and validating that it caused inhibition of EndMT (*Figure 1A*). In Phase 2, we evaluated the efficacy of EndMT inhibition via *SMAD3* knockdown to improve AVF patency (*Figure 1B*).

### 3.2 Creation and pilot validation of a novel preclinical pig AVF model (Phase 0)

In brief, we developed a preclinical large animal AVF model in pigs by anastomosing the ligated end of the femoral artery to the side of the femoral vein (see Supplementary material online, *Figure S1*). In Phase 0 of this study, we assessed the extent of EndMT in this model by comparing the venous limb of the AVF to the untouched contralateral femoral vein. From our prior studies in a mouse vein graft model, we had identified that the proportion of endothelial cells undergoing EndMT was substantial at ∼2 weeks after surgery.^14^ We therefore created an AVF in three pigs, and after 15 days, once AVF patency was confirmed by angiography, pigs were sacrificed and both the AVF and the untouched (control) left femoral vein were harvested (see Supplementary material online, *Figure S2A*). After tissue processing and mounting, the extent of EndMT was assessed by evaluating the co-expression of a set of faithful endothelial and mesenchymal markers.^14,15,17,21^ As expected, in the untouched (control) left femoral vein, only rare cells were observed that co-expressed endothelial and mesenchymal markers, suggesting that EndMT does not play a major role in normal vein homoeostasis in adult pigs. However, compared with the untouched (control) left femoral vein from the same pigs, the venous limb of the AVF showed a significantly increased proportion of endothelial cells that were co-positive for the combinations of either CD31^+^SM22α^+^ or VE-Cad^+^αSMA^+^ (see Supplementary material online, *Figure S2B* and *C*). Consistent with prior studies in mice,^14,17,18^ the proportion of co-positive cells undergoing EndMT ranged from ∼20 to 30%.

### 3.3 Evaluation of *SMAD3* knockdown efficiency and EndMT inhibition (Phase 1)

We next sought to develop and validate a robust gene delivery system to achieve local *in vivo SMAD3* knockdown in our AVF model. We tested lentiviral particles containing shRNAs directed against *SMAD3 in vitro* and identified a specific lentiviral construct that achieved an ∼90% knockdown of *SMAD3* in cultured porcine endothelial cells (see Supplementary material online, *Figure S3A*). In *ex vivo* studies using freshly harvested pig femoral veins, we identified that a dwell time of 60 min was sufficient to achieve lentiviral transfection of endothelial cells (see Supplementary material online, *Figure S3B*). Returning to our AVF model, we developed and optimized a protocol for injection and dwelling of the lentiviral construct (containing either shRNA against *SMAD3* or scrambled control shRNA) (see Supplementary material online, *Figures S1* and *S3C* and *Figure 4A*). In brief, once a section of the femoral artery and vein were dissected free, the proximal and distal ends of a 1.5-inch segment of the femoral vein were temporarily occluded and gently irrigated to flush out residual blood. Lentivirus was then administered as a single-dose intra-vessel injection and allowed to dwell in situ for 60 min. After this time, the segment of occluded vein was aspirated and gently flushed with saline to remove any residual viral article, and the AVF was created.

**Figure 2:**
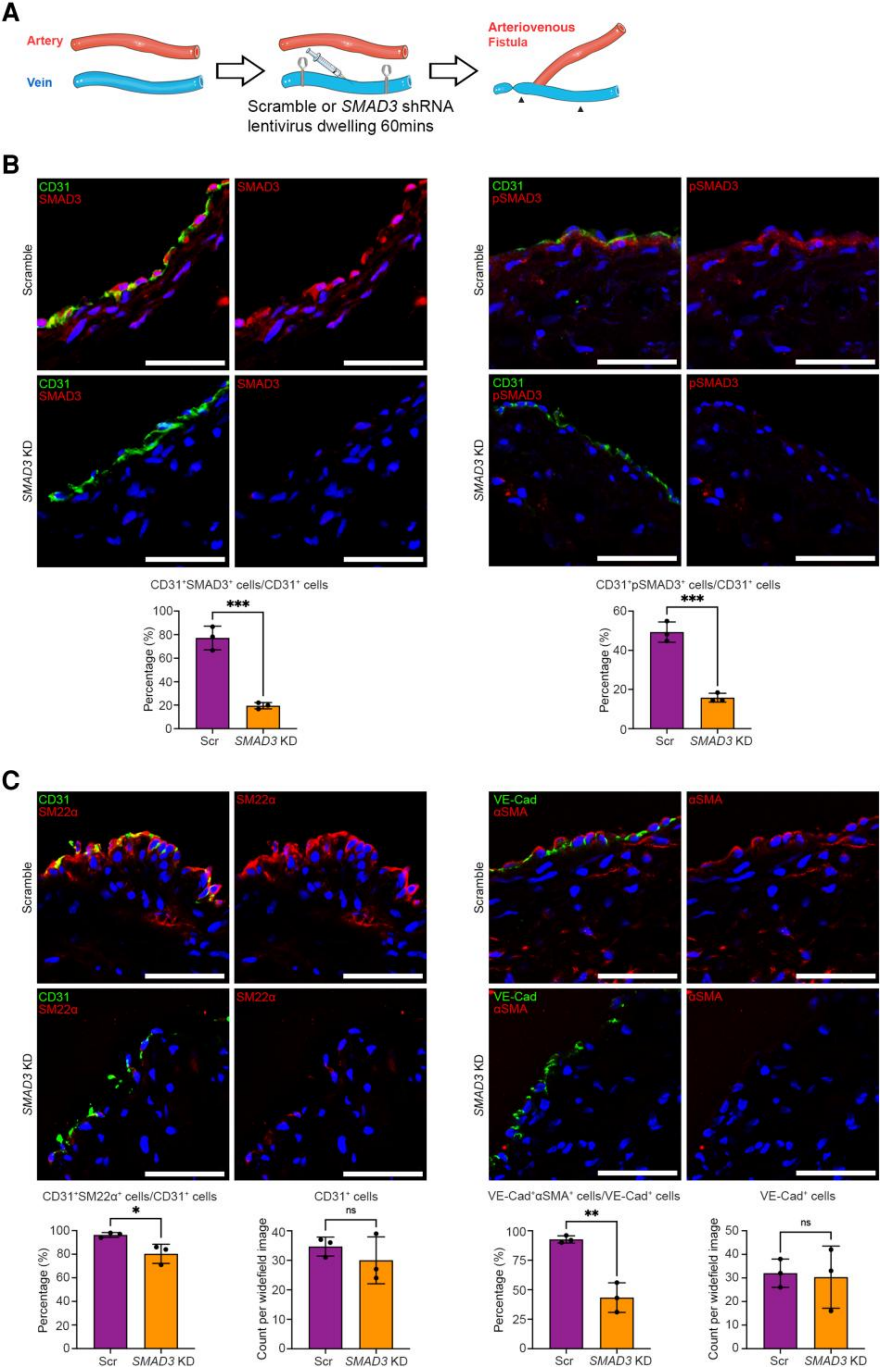
Efficacy of *SMAD3* knockdown and inhibition of EndMT in preclinical large animal AVF model (Phase 1). (*A*) Schematic representation of the surgical method including dwelling of lentivirus. Here, in Phase 1, the AVF in the right leg was harvested from six pigs 8 days after AVF creation, where three pigs were randomized to receive lentivirus carrying scramble shRNA (Scr; controls) and three pigs received lentivirus carrying *SMAD3* shRNA (*SMAD3* KD). Arrowheads indicate the section of vein where the lentivirus was allowed to dwell. (*B*) Representative immunofluorescence staining and quantitation of SMAD3 and pSMAD3 in endothelial cells 8 days after AVF creation. CD31 is shown in green, SMAD3 or pSMAD3 in red, and DAPI in blue. (*C*) Representative immunofluorescence staining and quantitation of EndMT and luminal endothelial cell coverage, 8 days after AVF creation. Endothelial markers (CD31 and VE-Cad) are shown in green. Mesenchymal markers (SM22α and αSMA) are in red. DAPI-stained nuclei are in blue. Analyses were performed using unpaired Student’s *t*-test. All scale bars = 50 µm. *P < 0.05; **P < 0.01; ***P < 0.001. *n* = 3 pigs per group for all analyses.

In Phase 1 of the study, six pigs were studied using the above protocol, with harvesting at 8 days after AVF creation to assess the effectiveness of *SMAD3* knockdown (*Figure 2B*) and inhibition of EndMT (*Figure 2C*). During the surgical procedure to create the AVFs, three pigs were randomized to receive lentivirus containing *SMAD3* shRNA (*SMAD3* knockdown), while three pigs received the same lentivirus containing scramble shRNA (controls). Confirming the importance of the TGF-β pathway and SMAD3 signalling during vein graft remodelling and the EndMT process, in the venous limb of control AVFs at this 8-day time point, we observed robust expression of both SMAD3 and phospho-SMAD3 (pSMAD3) in endothelial cells (77.2 ± 10.0% and 49.3 ± 5.1% of CD31^+^ cells expressed SMAD3 and pSMAD3, respectively; *Figure 2B*). Compared with the control group, the *SMAD3* knockdown group exhibited an ∼75% reduction in the proportion of CD31^+^ endothelial cells that expressed SMAD3 (77.2 ± 10.0 vs. 19.5 ± 2.7%, *P* < 0.001) and a 68% reduction in the proportion of CD31^+^ cells that expressed pSMAD3 (49.3 ± 5.1 vs. 15.8 ± 2.3%, *P* < 0.001) (*Figure 2B*). We also confirmed that *SMAD3* knockdown does not affect the level of SMAD2 or pSMAD2 within the endothelium (see Supplementary material online, *Figure S4*). In addition, compared with the venous limb of control AVFs, the *SMAD3* knockdown group showed a significant reduction in the extent of EndMT, as again assessed by the proportion of endothelial cells that were co-positive for the combinations of either CD31^+^SM22α^+^ or VE-Cad^+^αSMA^+^ (*Figure 2C*). The luminal endothelial cell coverage, however, was comparable in both groups at this 8-day time point, indicating that the *SMAD3* shRNA-containing lentivirus did not disturb endothelial cells in the acute phase (*Figure 2C*). These findings validate that *SMAD3* can be effectively knocked down by dwelling *SMAD3* shRNA-containing lentivirus for 60 min in the femoral vein immediately before the creation of a femoral AVF and that *SMAD3* knockdown inhibits EndMT.

### 3.4 Effect of EndMT inhibition by *SMAD3* knockdown in a preclinical AVF model (Phase 2)

**Figure 3:**
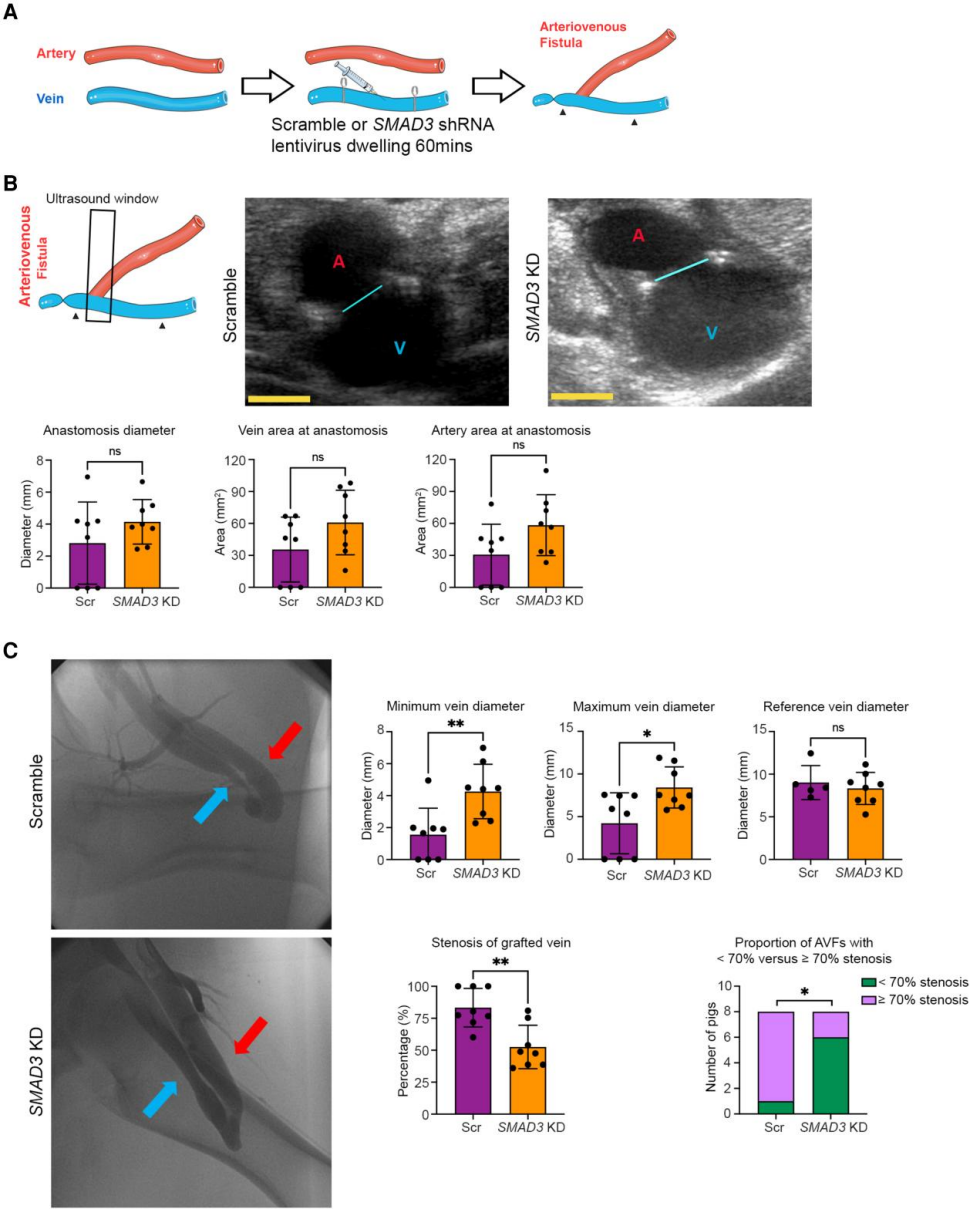
Ultrasound and angiographic evaluation of the efficacy of EndMT inhibition by *SMAD3* knockdown in preclinical large animal AVF model at 30 days (Phase 2). (*A*) Schematic representation of the surgical method including dwelling of lentivirus. Here, in Phase 2, the AVF in the right leg was harvested from 16 pigs 30 days after AVF creation, where eight pigs were randomized to receive lentivirus carrying scramble shRNA (Scr; controls) and eight pigs received lentivirus carrying *SMAD3* shRNA (*SMAD3* KD). Arrowheads indicate the section of vein where the lentivirus was allowed to dwell. (*B*) Ultrasound measurement at 30 days after AVF creation to assess the surgical anastomotic site and equivalence of AVF creation between groups. The left schematic image represents the orientation of the ultrasound probe during scanning with respect to the AVF. Arrowheads indicate the section of vein where the lentivirus was allowed to dwell. The black frame indicates the ultrasound scanning window. The middle and right panels show representative ultrasound images from scramble (control) and *SMAD3* knockdown pigs, respectively. The cyan line indicates the diameter of anastomosis; A in red colour indicates artery; V in blue colour indicates vein. Shown in the panels below are ultrasound quantifications of the anastomosis diameter, vein area and artery area (as acquired at the anastomosis site in the image plane as shown). Yellow scale bar = 5 mm. Anastomosis diameter and artery area were compared using unpaired Student’s *t*-test, while vein area was compared using a Mann–Whitney test. (*C*) Angiographic measurement of AVF diameter, stenosis, and patency 30 days after creation. Left panels show representative femoral angiography images. Red arrow indicates artery (arterial limb of AVF) and blue arrow indicates vein (venous limb of AVF). Corresponding diameters and stenosis severity of the venous limb of the AVF are presented on the right. ‘Minimum vein diameter’ represents the minimum diameter of the lentivirus-treated segment of the venous limb of the AVF, ‘maximum vein diameter’ represents the maximal diameter of the lentivirus-treated segment of the venous limb of the AVF, while ‘reference vein diameter’ represents the diameter of the reference vein segment from the adjacent untreated portion of the vein (cranial from the site of lentivirus dwelling). ‘Stenosis of grafted vein’ represents the stenosis of the lentivirus-treated segment of the venous limb of the AVF (determined by comparing the minimum with the reference diameters) presented as either % stenosis or the proportion with stenosis <70% vs. ≥70%. Minimum and maximum vein diameters were compared using Mann–Whitney test. Reference vein diameter and % stenosis of grafted vein were compared using unpaired Student’s *t*-test. Stenosis of grafted vein (<70% vs. ≥70%) was compared using Fisher’s exact test. *P < 0.05; **P < 0.01; ns, not significant. *n* = 8 pigs per group for all analyses except ‘reference vein diameter’ in *C* where *n* = 5 in the control group only (it was not possible to determine the reference vein diameter in the three occluded AVFs in the control group and therefore these are not presented).

Sixteen pigs that were randomized to receive either lentivirus containing *SMAD3* shRNA or lentivirus containing scramble shRNA (control) at the time of AVF creation (eight per group), and which were free of procedure-related complications, underwent analysis and are presented here (*Figures 1B* and *3A*). Terminal harvest of these 16 animals and the AVFs was on Day 30 after AVF creation. During the 30-day period of Phase 2, we did not note any obvious signs that certain animals were doing any better (or worse) than others from a systemic or overall perspective. Furthermore, at the time of tissue harvesting and euthanasia on Day 30, the body weights of the pigs were identical between groups. In addition, in both the *SMAD3* knockdown and control groups, pigs gained an average of 8 kg in the 30 days from AVF creation and dwelling of lentivirus to the terminal harvest time point (see Supplementary material online, *Figure S5*).

On Day 30 after surgery, ultrasound of the AVF was performed to evaluate patency and the size of the anastomosis (*Figure 3B*). We observed that three of the AVFs were totally occluded, which were subsequently identified as all being from the control group. Despite this, there was no difference in anastomosis size and vessel lumen area at the surgical anastomosis site between groups, confirming that the AVFs in the two groups were created equally (*Figure 3B*).

To further evaluate the effect of EndMT inhibition by *SMAD3* knockdown in our AVF model, the diameter and other features of the venous limb of the AVF were measured by femoral angiography (*Figure 3C* and Supplementary material online, *Figure S6*). We confirmed the ultrasound observation that three of the control AVFs were totally occluded, whereas all eight AVFs in the *SMAD3* knockdown group were patent. By angiography, the minimum diameter of the venous limb of the AVF was 1.56 ± 1.66 mm vs. 4.26 ± 1.71 mm for the control vs. *SMAD3* knockdown groups, respectively (*P* < 0.01), while the maximal diameter of the lentivirus-treated segment of the venous limb of the AVF was also greater in the *SMAD3* knockdown group (4.22 ± 3.58 vs. 8.42 ± 2.41 mm, respectively; *P* < 0.05). There was no difference in the mean diameter of the reference vein segments from the adjacent untreated portion of the vein (cranial from the site of lentivirus dwelling). Correspondingly, the mean % stenosis of the venous limb of the AVF was 83.30 ± 15.08% vs. 52.52 ± 16.99% for the control vs. *SMAD3* knockdown groups, respectively (*P* < 0.01) (*Figure 3C*). Moreover, in the control group, seven out of eight pigs developed stenosis of the venous AVF limb of ≥70% (which is similar to prior AVF studies in pigs^25^), while only two out of eight AVFs in the *SMAD3* knockdown group developed this degree of stenosis (*P* < 0.05) (*Figure 3C*).

**Figure 4:**
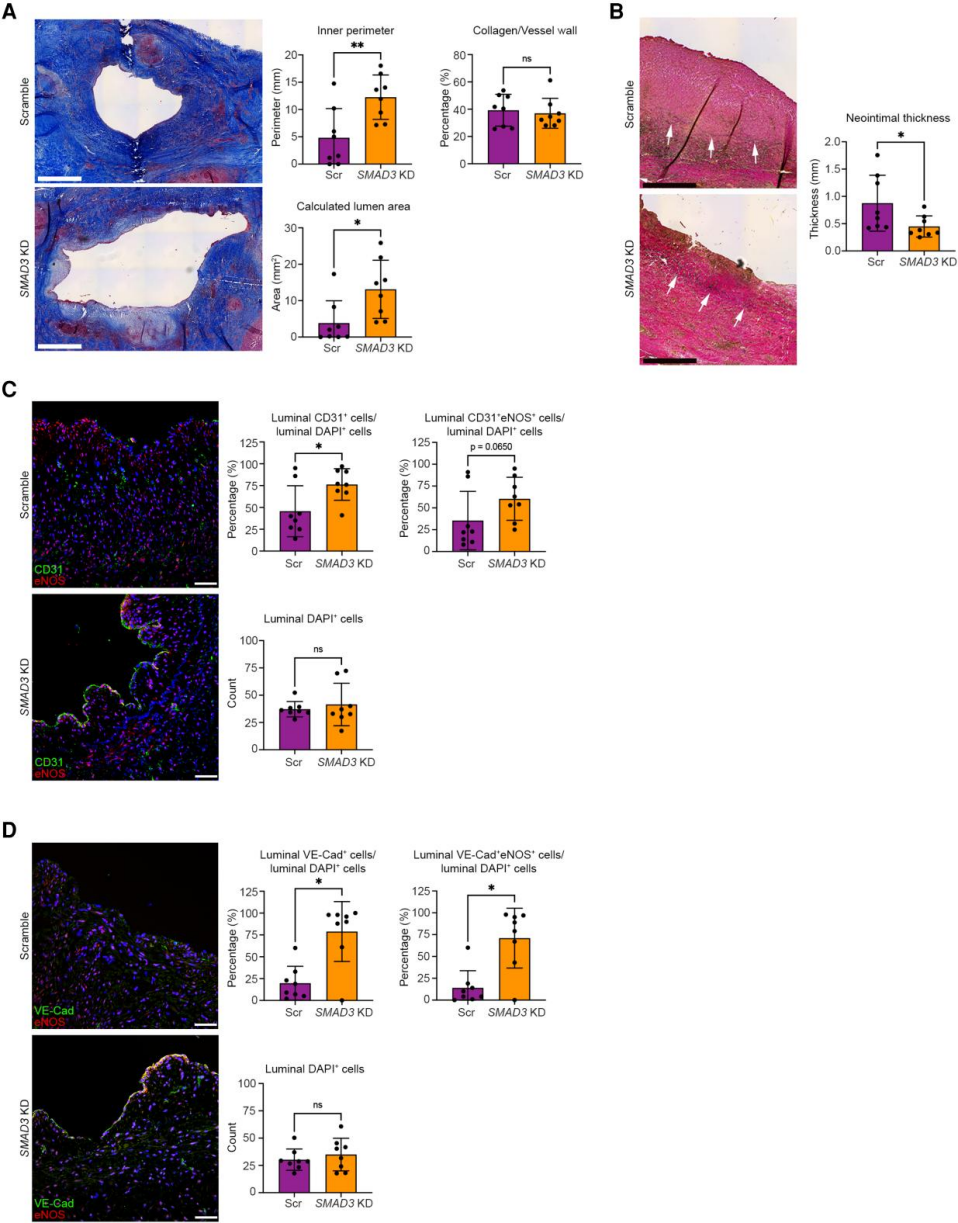
Histologic and immunofluorescence evaluation of the efficacy of EndMT inhibition by *SMAD3* knockdown in preclinical large animal AVF model at 30 days (Phase 2). (*A*) Representative images stained using Masson’s trichrome stain with analyses of inner perimeter, calculated lumen area, and collagen content of the vessel wall. Lumen area was calculated from the inner perimeter (i.e. inner circumference) and assuming the vessel was circular in cross-section. For this panel, all images are from the narrowest portion of the venous limb of the AVF. Scale bar = 1 mm. (*B*) Representative images stained using EVG stain to identify the inner elastic lamina that demarcates the intima-media boundary (arrows),^24^ with quantitation of overall neointimal thickness (from the intima-media boundary to the intima) for each AVF determined by averaging the neointimal thickness measurement from three sites per AVF from a single section. For *B*, images are from close to the narrowest portion of the venous limb of the AVF (within 1–2 mm). Scale bar = 0.5 mm. (*C*) Representative immunofluorescence staining for CD31 (green), eNOS (red), and DAPI-stained nuclei (blue) with quantifications. Scale bar = 50 µm. (*D*) Representative immunofluorescence staining for VE-Cad (green), eNOS (red), and DAPI-stained nuclei (blue) with quantifications. Scale bar = 50 µm. Images in *C* and *D* are from the venous limb of the AVF, within 5–10 mm of the narrowest portion. Analyses were performed as follows: (*A*) inner perimeter with unpaired Student’s *t*-test and both calculated lumen area and collagen content with Mann–Whitney test; (*B*) neointimal thickness with unpaired Student’s *t*-test; (*C*) CD31^+^ cells/DAPI^+^ cells and DAPI^+^ cells with unpaired Student’s *t*-test, CD31^+^eNOS^+^/DAPI^+^ cells with Mann–Whitney test; (*D*) DAPI^+^ cells with unpaired *t*-test; other analyses in *D* with Mann–Whitney test. *P < 0.05; **P < 0.01; ns, not significant. *n* = 8 pigs per group for all analyses.

Histopathological analysis of the venous limbs of these AVFs comparing the control vs. *SMAD3* knockdown groups confirmed significant increases in both the inner lumen perimeter (i.e. circumference; 4.83 ± 5.35 vs. 12.26 ± 4.07 mm, respectively; *P* < 0.01) and the calculated lumen area (3.84 ± 6.12 vs. 13.12 ± 8.00 mm^2^, respectively; *P* < 0.05), but with no difference in collagen content of the vessel wall (*Figure 4A*). Correspondingly, as compared with controls, neointimal thickness was significantly reduced in veins from the *SMAD3* knockdown group (0.88 ± 0.51 vs. 0.45 ± 0.19 mm, respectively; *P* < 0.05) (*Figure 4B*).

Because inhibition of EndMT is expected to preserve the endothelial phenotype and thus potentially improve endothelial integrity and AVF endothelialization, we evaluated the proportion of luminal cells that expressed CD31 and endothelial NO synthase (eNOS). Comparing the venous limbs of the AVFs between the control vs. *SMAD3* knockdown groups, we observed improved endothelial integrity as determined by the proportion of luminal cells that expressed CD31 (*Figure 4C*). In addition, there was a borderline increase in the proportion of CD31^+^eNOS^+^ co-positive endothelial cells (*P* = 0.065; *Figure 4C*). Using VE-Cad as an alternate marker for endothelial cells, similar results were observed, with a significant increase in both the proportion of luminal cells that expressed VE-Cad and also the proportion of VE-Cad^+^eNOS^+^ co-positive endothelial cells in the *SMAD3* knockdown group (*P* < 0.05; *Figure 4D*).

**Figure 5:**
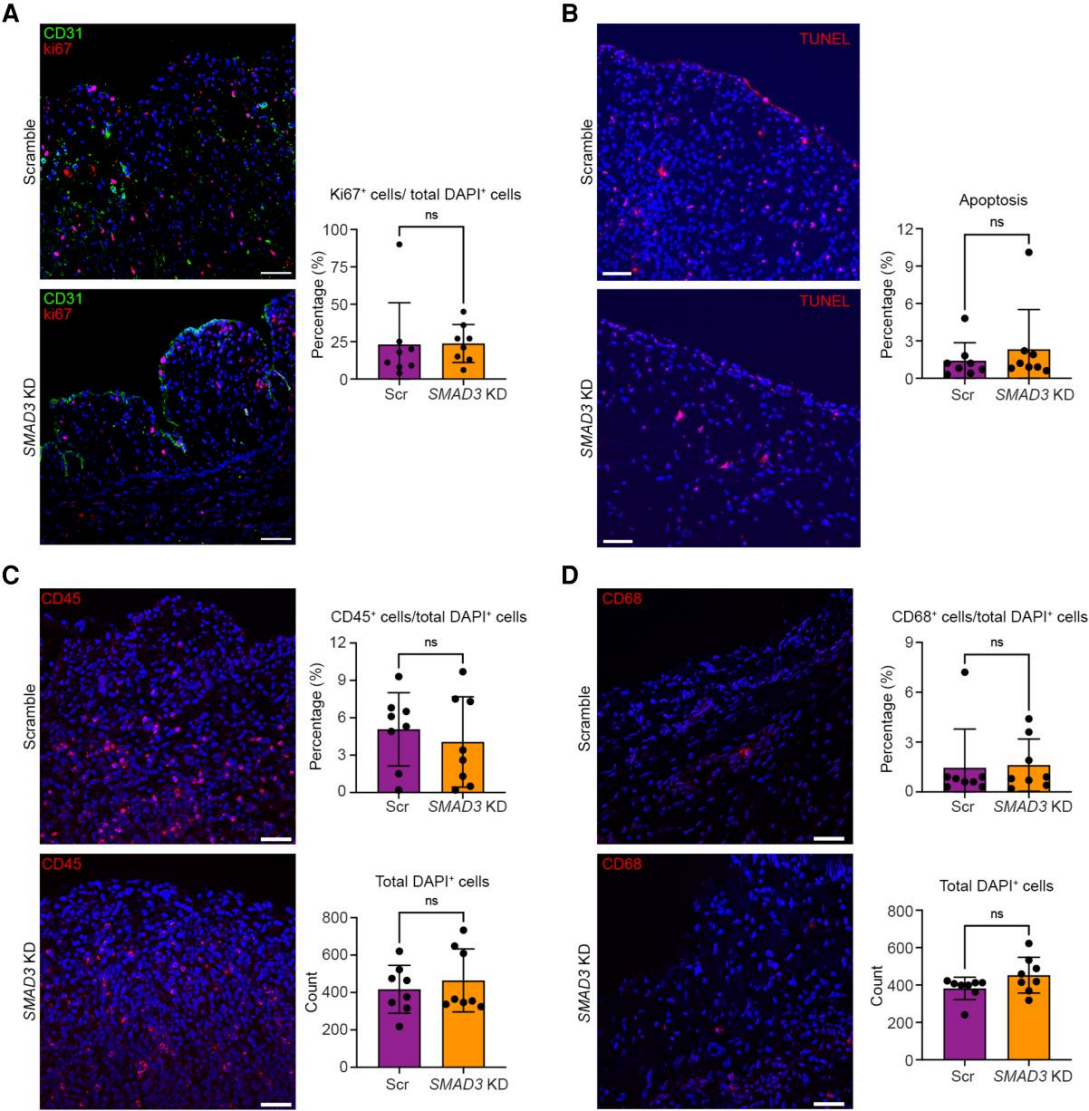
Evaluation of the effect of EndMT inhibition by *SMAD3* knockdown on cell proliferation, apoptosis, and immune cell infiltration in a preclinical large animal AVF model at 30 days (Phase 2). Images in this figure are from the venous limb of the AVF, within 5–10 mm of the narrowest portion. (*A*) Assessment of cell proliferation with representative immunofluorescence staining for CD31 (green), Ki67 (red), and DAPI-stained nuclei (blue) with quantifications. (*B*) Assessment of apoptosis with representative immunofluorescence staining for TUNEL assay (red) and DAPI-stained nuclei (blue) with quantifications. (*C*) Assessment of immune cell infiltration with representative immunofluorescence staining for CD45 (red) and DAPI-stained nuclei (blue) with quantifications. (*D*) Assessment of immune cell infiltration with representative immunofluorescence staining for CD68 (red) and DAPI-stained nuclei (blue) with quantifications. All analyses were performed using Mann–Whitney test except for in *C*; CD45+ cell/total DAPI analysis was performed with unpaired Student’s *t*-test. All scale bars = 50 µm. ns, not significant. *n* = 8 pigs per group for all analyses.

To investigate other aspects that may have been of relevance, we compared cell proliferation (Ki67^+^ cells; *Figure 5A*), apoptosis (TUNEL assay; *Figure 5B*), and immune cell infiltration (CD45^+^ and CD68^+^ cells; *Figure 5C* and *D*, respectively) in the venous limb of AVFs between the control vs. *SMAD3* knockdown groups. There were no differences observed for any of these parameters.

Because the pathological processes may differ between patent AVFs and those with complete occlusion, we re-analysed the findings in *Figures 3*–*5* after removing data related to the three AVFs from the control group that were totally occluded. Although there was reduced statistical power, the results were similar (see Supplementary material online, *Figures S7*–*S9*).

## 4. Discussion

There has been intense recent interest in the possibility that inhibition of EndMT may be a viable clinical approach to treat various cardiovascular diseases.^15,16,20,21^ However, despite many positive studies in small animals, there have been no dedicated translational attempts to investigate this possibility in a large animal model.^15,16,20,21^ Here, based on our earlier studies in mice,^14^ we created a large animal AVF model and investigated the effect of EndMT inhibition by local knockdown of *SMAD3*, with a specific view towards understanding the likely clinical utility of this approach. Using a range of different readouts that included immunofluorescence staining, histopathology, angiography, and ultrasound, our results were consistent in showing that the inhibition of EndMT leads to a marked improvement of luminal dimensions, while neointimal formation and stenosis severity were both reduced (*Figures 3* and *4*; *Graphical Abstract*). Furthermore, the inhibition of EndMT was associated with improved endothelialization and an increase in the expression of eNOS (*Figure 4C* and *D*). This latter finding may be of importance, as eNOS levels are known to be reduced in the setting of EndMT,^17,26^ with eNOS known to play a central role in the tuning and maintenance of endothelial homoeostasis.^27^ Collectively, these data suggest that this therapeutic approach holds significant promise for use in humans undergoing AVF and potentially any vein graft surgery.

A key aspect of our study from the perspective of human translation is our simple and efficient system for local *in vivo SMAD3* knockdown, which entirely avoids any dissemination or systemic administration of the therapeutic agent. This involved very gentle occlusion of the segment of the vein that will become the venous limb of the AVF using human surgical vascular techniques and instruments, then using a fine gauge catheter the local blood was aspirated and the vein segment gently flushed with saline. The lentiviral construct was then gently injected and allowed to dwell. After 60 min, the lentiviral article was aspirated out of the occluded vein, followed by a final gentle saline flushing and creation of the AVF. Although these simple steps will add ∼65 min to the procedure time required to create an AVF in humans (including 60 min for lentiviral dwelling), it will require minimal or perhaps even no additional surgical training to implement in the clinic. This approach is also readily adaptable to all other types of human vein graft surgery. While a theoretical concern of our approach involving local *SMAD3* knockdown might be outward expansion and pseudo-aneurysm formation, the data on lumen perimeter and area in our study (*Figures 3* and *4*) provide assurance that this does not occur in this model.

In terms of the optimal method to achieve *SMAD3* knockdown, we chose a lentiviral system because they are efficient and highly effective for transducing endothelial cells (see Supplementary material online, *Figure S3*). While adenoviruses and AAVs are often favoured because they typically do not integrate into the genome of the host cell, endothelial cells are highly resistant to transduction by unmodified AAVs.^22^ However, a long-standing concern regarding lentiviral and other retroviral systems is the risk of insertional oncogenesis, whereby the lentivirus or retrovirus (which permanently integrates into host DNA) could insert into the promoter region of an oncogene, thereby activating that oncogene and causing malignant cell transformation. However, this concern appears to have been largely overcome by advances in gene therapy technologies, whereby lentiviral integration is now understood to largely occur in intronic regions, not promoter sites.^28^ Furthermore, if lentiviral approaches were used in humans during AVF creation or other vein graft surgery, the approach would be strictly local. Thus, any theoretical risk of insertional oncogenesis would be limited to the treated segment of the vein. Signifying the advantages of lentiviral approaches, it is exciting to note that two lentiviral gene therapy products have recently been licensed for human use in the USA.^28^

Several additional aspects of this study warrant comment with respect to the human translational potential of this approach. Notably, the extent of EndMT was striking in our large animal AVF model, both at the 8-day (*Figure 2*) and 15-day (see Supplementary material online, *Figure S2*) time points. Providing proof of the clinicopathological relevance of EndMT in humans has been a challenge for the field.^15^ This has largely been due to difficulties in acquiring relevant human tissues at the critical time points that are required to study EndMT. For example, it is not typically possible to obtain human tissues following successful AVF creation, as we were able to do in this study. Our finding of prominent EndMT in this large animal model provides important additional translational insight because it corroborates murine data^14^ and confirms that EndMT is a viable clinical target.

While our study is the first to establish a specific large animal model for the ultimate purpose of targeting EndMT in humans, others have previously identified EndMT in differing large animal settings. For example, Dal-Bianco *et al*.^29^ showed that mitral valve endothelial cells undergo EndMT in a mitral valve regurgitation model in sheep. They also showed that losartan can reduce the extent of EndMT and the thickness of the mitral valve leaflets in a combined model of both myocardial infarction and mitral valve leaflet tethering.^30^ However, the potential clinical utility of an isolated reduction in the thickness of the mitral valve leaflets as a means to reduce subsequent leaflet fibrosis in this setting is unknown. As other examples of large animal studies, Hong *et al*.^31^ showed that the induction of iliac vein compression in a pig model induces EndMT, which may contribute to local iliac vein thrombosis. However, there was not any therapeutic manipulation of EndMT in pigs by these investigators. Finally, other research groups have documented that EndMT occurs in pigs,^32–34^ but without any attempt to inhibit or manipulate EndMT in pig models.

This study was conceived and designed to primarily address the translational and clinical potential of EndMT inhibition by *SMAD3* knockdown for human vein grafting and AVF creation. Our decision to focus this study on the potential clinical impact of this approach was partly motivated by our prior publication in a mouse model of vein grafting, where we undertook in-depth molecular analyses of the key mechanisms of Smad3 inhibition and EndMT.^14^ Specifically, in that study, we showed that EndMT contributes to neointimal formation in mice during vein graft remodelling, which is dependent on early activation of the Smad2/3-Slug signalling pathway. Antagonism of TGF-β signalling by neutralizing antibody, short hairpin RNA-mediated *Smad3* or *Smad2* knockdown, *Smad3* haploinsufficiency, or endothelial cell-specific *Smad2* deletion resulted in decreased EndMT and reduced neointimal formation compared with controls.^14^ Because, from that study, the role of Smad3 was already well understood in the context of vein graft remodelling in mice, it was a logical target for this large animal study. However, we acknowledge that other factors and pathways also govern EndMT, including epigenetic mechanisms and other mediators such as HDAC9,^18^ SIRT1,^35^ and JMJD2B.^36^ These factors and alternate pathways may also hold clinical translational potential.

### 4.1 Limitations

Using pigs for our animal model, it is not possible to perform rigorous lineage tracking of endothelial cells as is possible in mice using Cre-lox systems. Rather, we assessed the extent of EndMT by co-expression of faithful endothelial and mesenchymal markers. This limits the detection of EndMT to where cells have retained their endothelial markers, and therefore, this method precludes the detection of EndMT-derived mesenchymal cells that have lost the expression of endothelial markers. As a result, we may have underestimated the extent of EndMT in this study. In addition, the range of antibodies available for use in pigs is much more limited than in mice and humans, which constrained our ability to undertake more extensive immunofluorescence analyses. As a further consideration, we used young healthy pigs that did not have renal failure, whereas most patients requiring an AVF for haemodialysis are middle-aged or older individuals with end-stage renal disease, often with other co-morbidities such as diabetes. Until our approach is evaluated in these settings, caution should be applied in considering the clinical utility of our findings.

### 4.2 Conclusions

The inhibition of EndMT has been proposed as a promising clinical therapeutic target,^15,16,20,21^ but until now, there have been no dedicated large animal studies performed with a specific view towards direct human translation. Based on prior murine studies,^14^ we developed a preclinical large animal AVF model and found substantial EndMT in this setting. The inhibition of EndMT using a *SMAD3* lentiviral construct at the time of AVF creation was associated with a significant reduction in neointimal hyperplasia, increased endothelialization, and a reduction in the degree of AVF stenosis at 30 days. These findings build on a large body of *in vitro* and *in vivo* rodent data and suggest that the local inhibition of EndMT by gene therapy is a promising approach for improving AVF and other vein graft patency in humans.

**Translational perspective**

By creating a femoral arteriovenous fistula (AVF) in pigs, we developed the first dedicated preclinical large animal model to inhibit endothelial-to-mesenchymal transition (EndMT). Inhibition of EndMT in this large animal model by *SMAD3* knockdown using gene therapy led to reduced neointimal hyperplasia, increased endothelialization, and a reduction in the degree of AVF stenosis. This study provides proof of concept for the inhibition of EndMT as a clinical strategy to improve the patency of AVFs (e.g. haemodialysis fistulas) and vein grafts. Our simple approach, using a targeted lentiviral gene therapy that is applied locally during AVF or vein graft surgery, could be easily implemented in the clinic. The study also provides broader proof of concept to continue the investigation of EndMT inhibition as a therapeutic approach for other cardiovascular diseases.

## Supplementary Material

cvae157_Supplementary_Data

## Data Availability

The data underlying this article will be shared on reasonable request to the corresponding author.
