# Supplementary material for: Inhibition of endothelial-to-mesenchymal transition in a large animal preclinical arteriovenous fistula model leads to improved remodelling and reduced stenosis
Source: Cardiovasc Res. 2024 Jul 26;120(14):1768–79. doi: 10.1093/cvr/cvae157 (PMC11587554; doi:10.1093/cvr/cvae157)
Supplement: cvae157_Supplementary_Data [file cvae157_supplementary_data.zip › 20240531 Pig EndMT Supplemental Figures (1).pdf]

Suppl. Fig 1

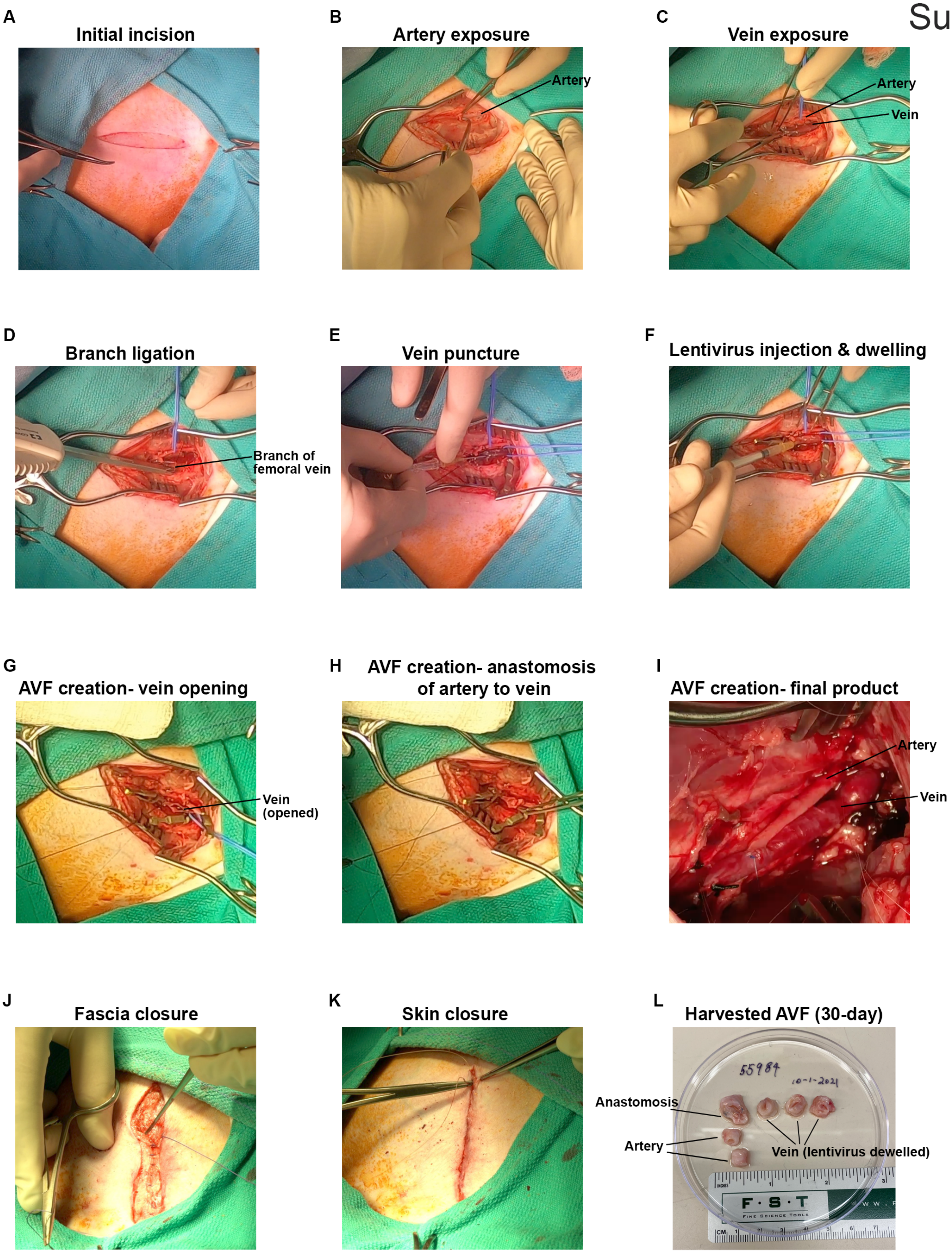
A
Initial incision
B
Artery exposure
Artery
C
Vein exposure
Artery
Vein
D
Branch ligation
Branch of femoral vein
E
Vein puncture
F
Lentivirus injection & dwelling
G
AVF creation- vein opening
Vein (opened)
H
AVF creation- anastomosis of artery to vein
I
AVF creation- final product
Artery
Vein
J
Fascia closure
K
Skin closure
L
Harvested AVF (30-day)
55984
10-1-2021
Anastomosis
Artery
Vein (lentivirus dewelled)
F·S·T
FINE SCIENCE TOOLS

Suppl. Fig 2

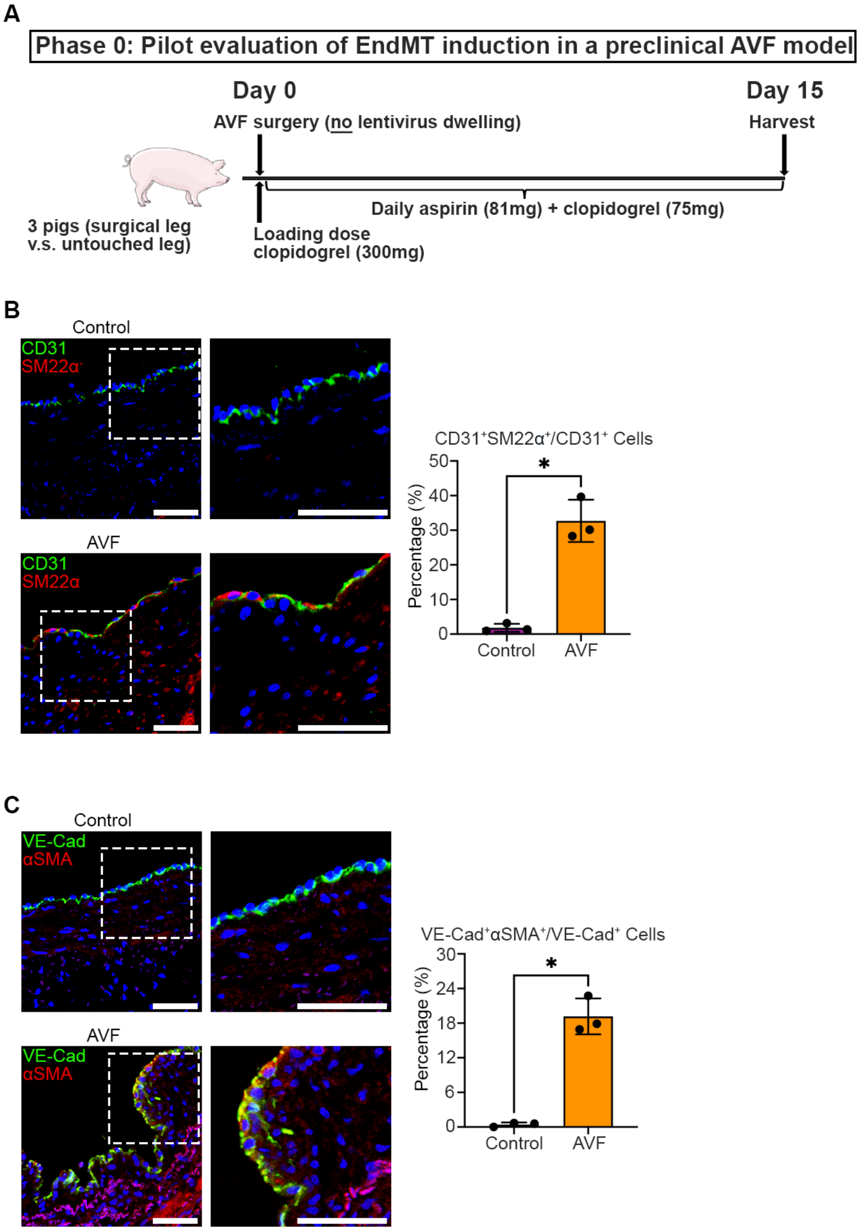
A
Phase 0: Pilot evaluation of EndMT induction in a preclinical AVF model
Day 0
AVF surgery (no lentivirus dwelling)
Day 15
Harvest
3 pigs (surgical leg v.s. untouched leg)
Loading dose clopidogrel (300mg)
Daily aspirin (81mg) + clopidogrel (75mg)
B
Control
CD31
SM22α
AVF
CD31
SM22α
CD31⁺SM22α⁺/CD31⁺ Cells
Percentage (%)
0
10
20
30
40
50
*
Control
AVF
C
Control
VE-Cad
αSMA
AVF
VE-Cad
αSMA
VE-Cad⁺αSMA⁺/VE-Cad⁺ Cells
Percentage (%)
0
6
12
18
24
30
*
Control
AVF

Suppl. Fig 3

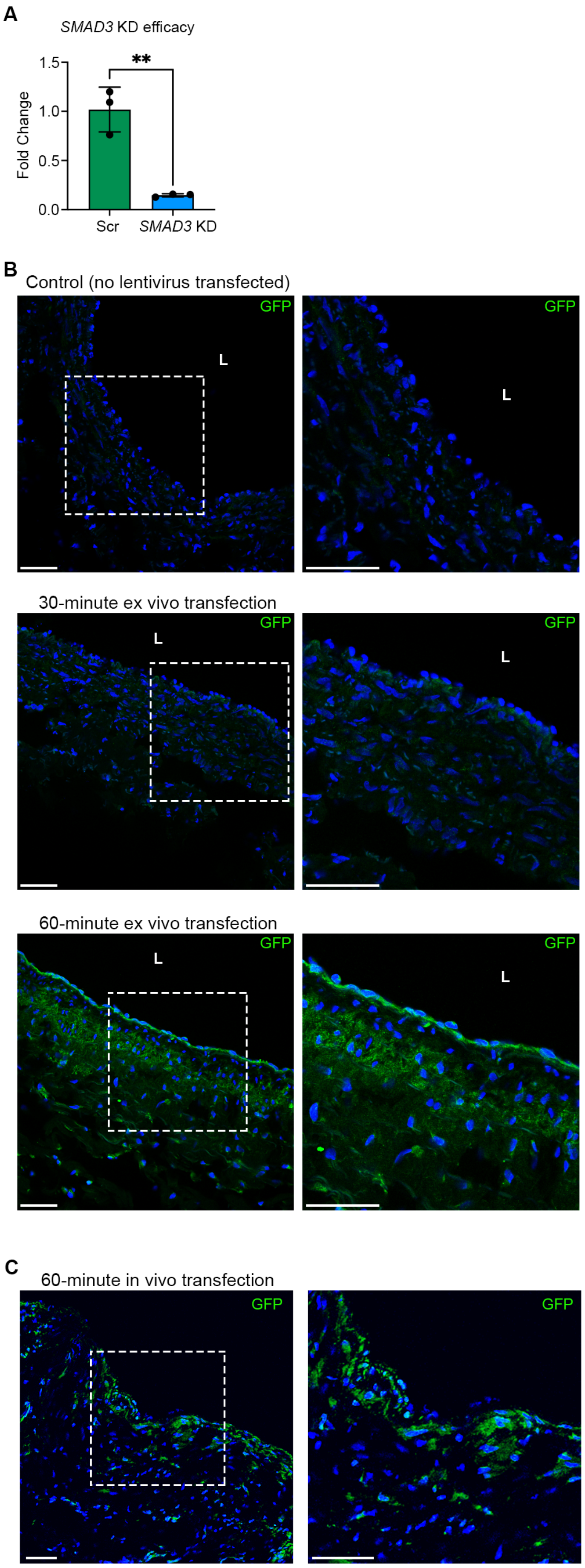
A
SMAD3 KD efficacy
**
1.5
1.0
0.5
0.0
Fold Change
Scr
SMAD3 KD
B
Control (no lentivirus transfected)
GFP
L
30-minute ex vivo transfection
60-minute ex vivo transfection
C
60-minute in vivo transfection

Suppl. Fig 4

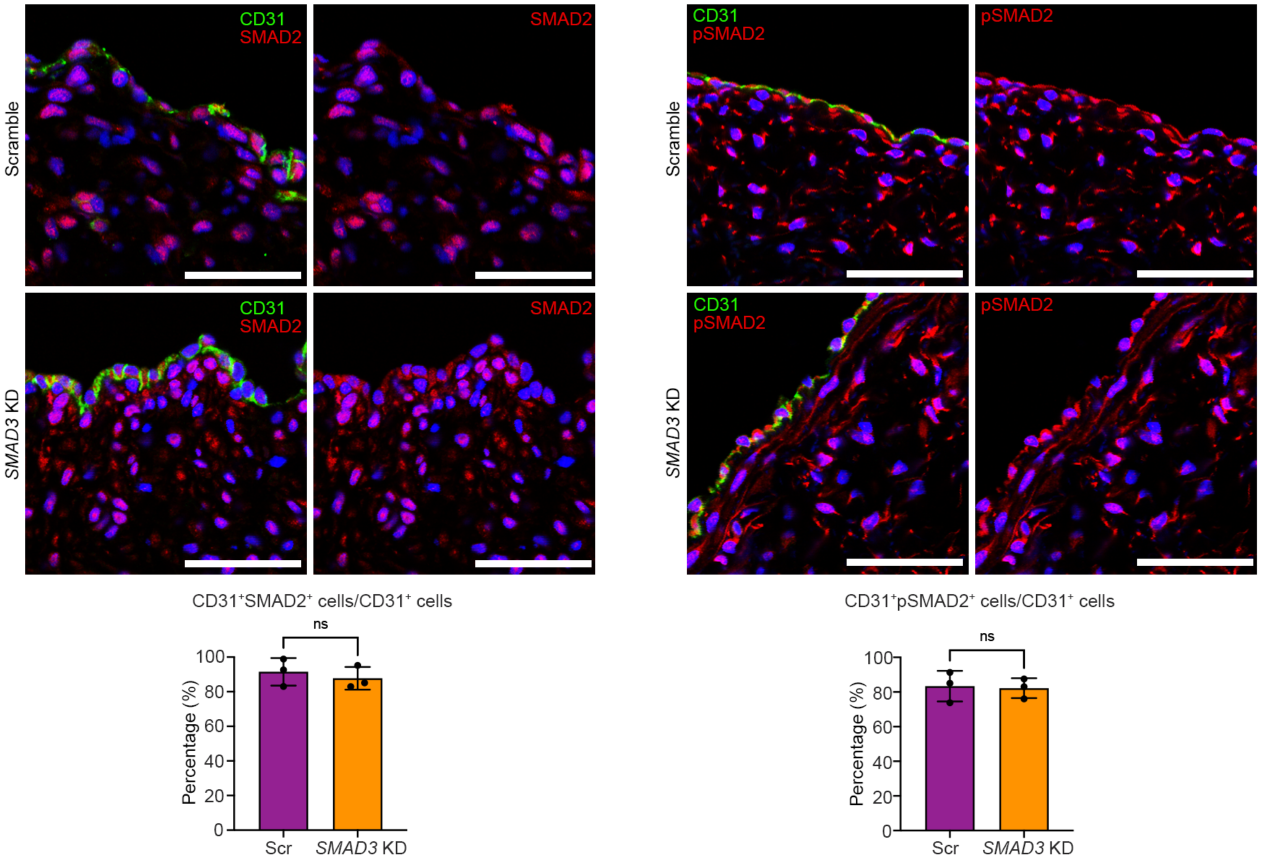

CD31
SMAD2
SMAD2
Scramble
SMAD3 KD
CD31
pSMAD2
pSMAD2
CD31+SMAD2+ cells/CD31+ cells
ns
Percentage (%)
100
80
60
40
20
0
Scr
SMAD3 KD
CD31+pSMAD2+ cells/CD31+ cells
ns
Percentage (%)
Scr
SMAD3 KD

Suppl. Fig 5

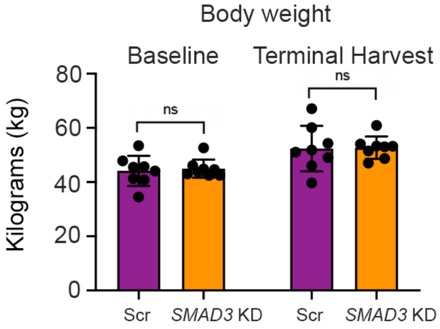

Body weight
Baseline
Terminal Harvest
Kilograms (kg)
80
60
40
20
0
ns
ns
Scr
SMAD3 KD
Scr
SMAD3 KD

Suppl. Fig 6

Control lentivirus

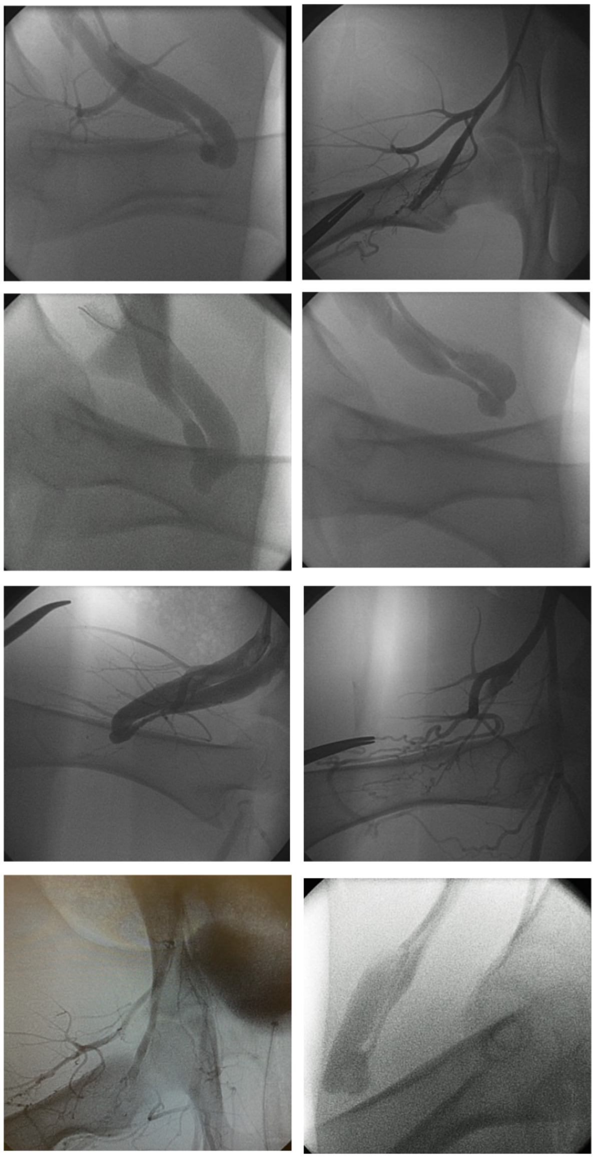

***SMAD3* knockdown lentivirus**

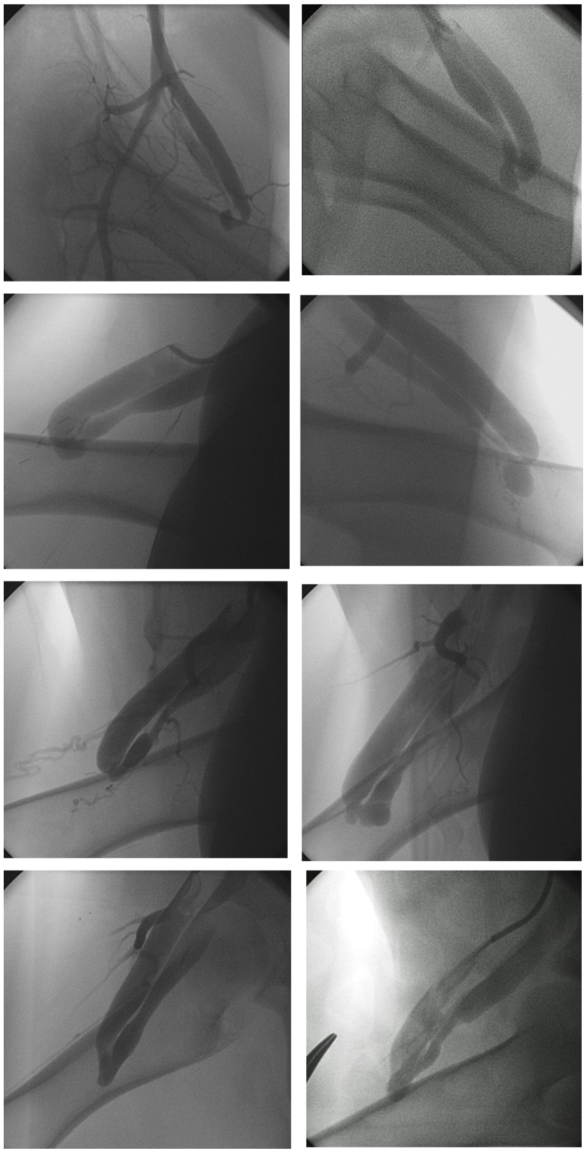

Suppl. Fig 7

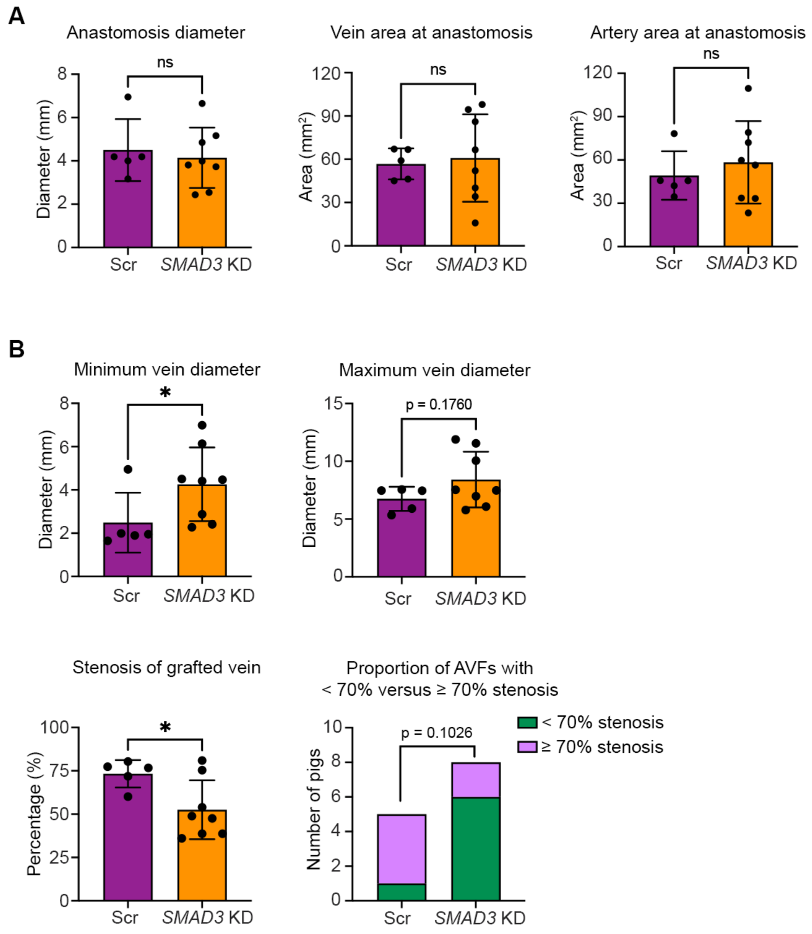
A
Anastomosis diameter
ns
Diameter (mm)
Scr
SMAD3 KD
Vein area at anastomosis
Area (mm²)
Artery area at anastomosis
B
Minimum vein diameter
*
Maximum vein diameter
p = 0.1760
Stenosis of grafted vein
Percentage (%)
Proportion of AVFs with
< 70% versus ≥ 70% stenosis
p = 0.1026
Number of pigs
< 70% stenosis
≥ 70% stenosis

Suppl. Fig 8

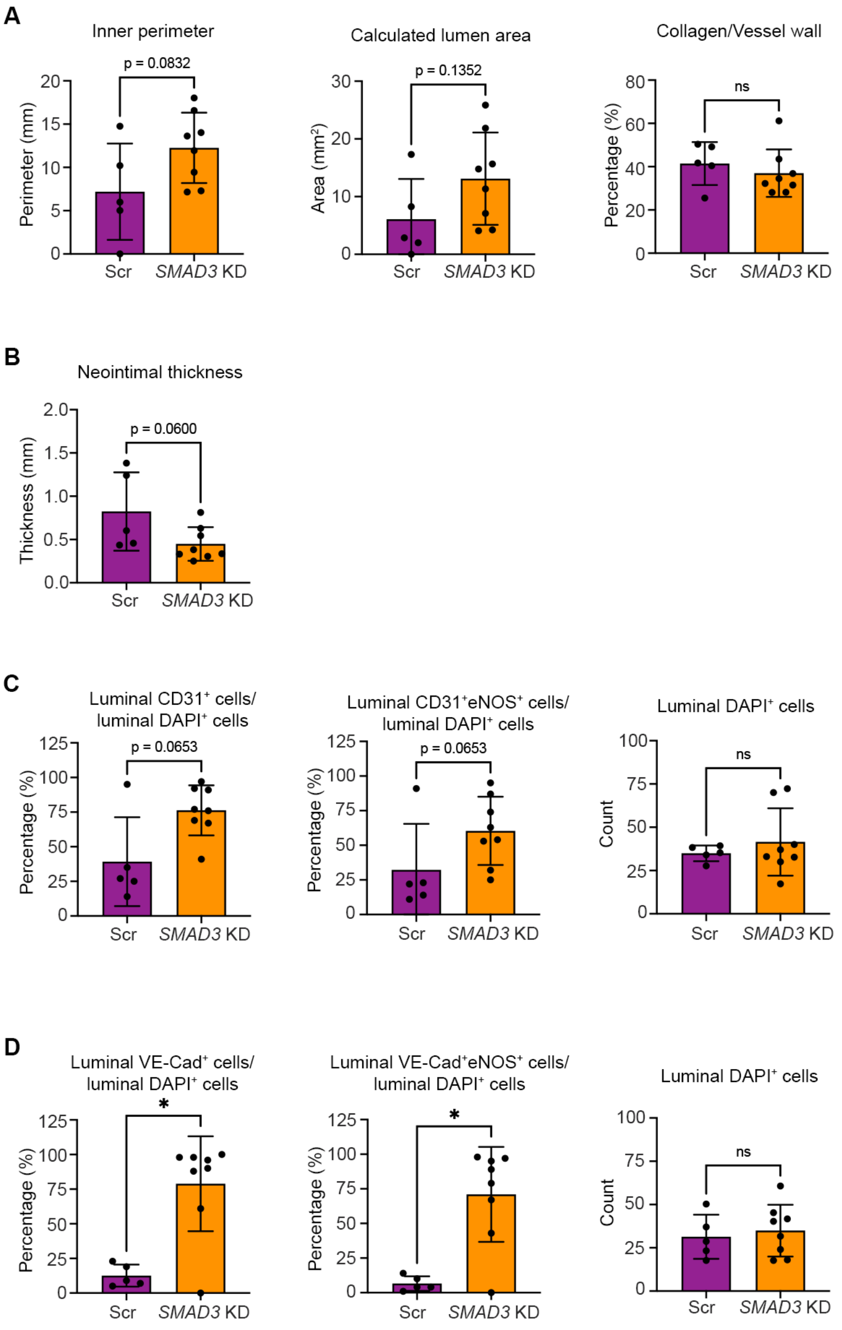
A
Inner perimeter
p = 0.0832
Perimeter (mm)
0
5
10
15
20
Scr
SMAD3 KD
Calculated lumen area
p = 0.1352
Area (mm²)
0
10
20
30
Scr
SMAD3 KD
Collagen/Vessel wall
ns
Percentage (%)
0
20
40
60
80
Scr
SMAD3 KD
B
Neointimal thickness
p = 0.0600
Thickness (mm)
0.0
0.5
1.0
1.5
2.0
Scr
SMAD3 KD
C
Luminal CD31⁺ cells/
luminal DAPI⁺ cells
p = 0.0653
Percentage (%)
0
25
50
75
100
125
Scr
SMAD3 KD
Luminal CD31⁺eNOS⁺ cells/
luminal DAPI⁺ cells
p = 0.0653
Percentage (%)
0
25
50
75
100
125
Scr
SMAD3 KD
Luminal DAPI⁺ cells
ns
Count
0
25
50
75
100
Scr
SMAD3 KD
D
Luminal VE-Cad⁺ cells/
luminal DAPI⁺ cells
*
Percentage (%)
0
25
50
75
100
125
Scr
SMAD3 KD
Luminal VE-Cad⁺eNOS⁺ cells/
luminal DAPI⁺ cells
*
Percentage (%)
0
25
50
75
100
125
Scr
SMAD3 KD
Luminal DAPI⁺ cells
ns
Count
0
25
50
75
100
Scr
SMAD3 KD

Suppl. Fig 9

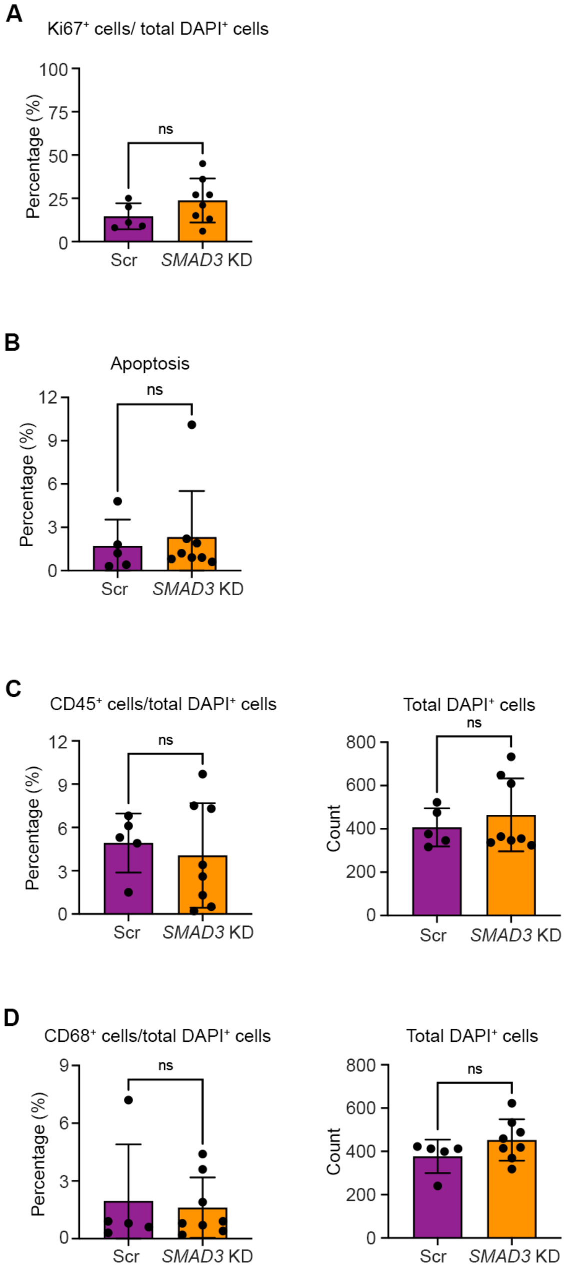
A
Ki67+ cells/ total DAPI+ cells
Percentage (%)
100
75
50
25
0
ns
Scr
SMAD3 KD
B
Apoptosis
Percentage (%)
12
9
6
3
0
ns
Scr
SMAD3 KD
C
CD45+ cells/total DAPI+ cells
Percentage (%)
12
9
6
3
0
ns
Scr
SMAD3 KD
Total DAPI+ cells
Count
800
600
400
200
0
ns
Scr
SMAD3 KD
D
CD68+ cells/total DAPI+ cells
Percentage (%)
9
6
3
0
ns
Scr
SMAD3 KD
Total DAPI+ cells
Count
800
600
400
200
0
ns
Scr
SMAD3 KD
