# Supplementary material for: Inhibition of endothelial-to-mesenchymal transition in a large animal preclinical arteriovenous fistula model leads to improved remodelling and reduced stenosis
Source: Cardiovasc Res. 2024 Jul 26;120(14):1768–79. doi: 10.1093/cvr/cvae157 (PMC11587554; doi:10.1093/cvr/cvae157)
Supplement: cvae157_Supplementary_Data [file cvae157_supplementary_data.zip › 20240606 Supplemental Material.docx]

**Supplemental Material**

***Inhibition of endothelial to mesenchymal transition in a large animal preclinical arterio-venous fistula model leads to improved remodeling and reduced stenosis***

**SUPPLEMENTAL METHODS**

*In vitro* evaluation of a lentiviral construct for *SMAD3* knockdown

Pig coronary artery endothelial cells (PCAEC; purchased as PP30005K from Genlantis, San Diego, CA; now sold as AMS.PP30005 by Amsbio, Cambridge, MA) were cultured at 37°C in 5% CO_2_ in custom media (purchased as PMP211500 from Genlantis; now sold as AMS.PMP211500 by Amsbio). To evaluate lentiviral constructs for knockdown of *SMAD3*, lentiviral particles containing differing strands of Santa Cruz’s *SMAD3* or scramble shRNA at 5 x 10^6^ IFU/ml were transfected into PCAECs using polybrene (sc-134220, Santa Cruz), according to the manufacturer’s instructions, with approximately 5000 IFU (10µl) applied to each well of a 6-well plate for 48 hours. Note that these Santa Cruz constructs were directed against human *SMAD3*, and in standard Santa Cruz orders *SMAD3* shRNA lentiviral particles (sc-38376-V) are provided as a pool of concentrated viral particles containing several target-specific constructs that encode 19-25 nt (plus hairpin) shRNA designed to knock down *SMAD3* gene expression. In studies using custom orders from Santa Cruz of lentiviral particles with the individual *SMAD3* shRNA strands, we identified a specific strand with robust activity against porcine *SMAD3* in PCAECs (Supplemental Figure 3A) and selected this specific strand for our ongoing studies.

To assess *SMAD3* knockdown efficacy *in vitro*, PCAECs were harvested after 48 hours of transfection and the level of *SMAD3* was determined by quantitative real time-polymerase chain reaction (qRT-PCR). In detail, RNA was first extracted using a RNeasy Mini Kit (#74104, QIAGEN, Germantown, MD) and quantified by a NanoDrop 2000c Spectrophotometer (Thermo Scientific, Waltham, MA). qRT-PCR was performed after obtaining cDNA using an iScript™ cDNA Synthesis Kit (1708891, Bio-Rad, Hercules, CA). Conditions for qRT-PCR were: 95°C for 5 minutes, 40 cycles of 95°C for 5 seconds and 60°C for 30 seconds. 18s rRNA was used as a control and gene expression analyzed using the ΔΔCt method. Primer sequences are provided in Supplemental Table 1.

Lentiviral construct for *ex vivo* and *in vivo* *SMAD3* knockdown in pigs

Following the above *in vitro* studies proving efficacy in PCAECs, the lentiviral construct used for all *ex vivo* and *in vivo* experiments in pigs was purchased from Santa Cruz Biotechnologies. This was a customized order and comprised a single human strand of *SMAD3* shRNA lentiviral particles, provided at high-titer (> 10^9^ IFU/ml). In this construct, *SMAD3* shRNA was under the control of an H1 promoter. The lentiviral sequence also contained GFP. Our control consisted of the identical lentiviral construct from Santa Cruz which contained a scramble shRNA sequence. In pilot studies, we determined that the GFP could only be visualized by using an anti-GFP antibody (Supplemental Figures 3B and 3C). Nevertheless, except for anti-GFP staining, all other immunofluorescence staining for this entire project was performed in fluorescence channels with excitation at 546nm and 633nm to avoid any potential confounding of this GFP signal.

Pig use and sex as a biological variable

Female Yorkshire pigs at approximately 40kg body weight were purchased from Animal Biotech Industries, Inc. (Doylestown, PA). Funding for this study was originally obtained via NIH grant R01HL130423, which was submitted and budgeted prior to the NIH policy on “Consideration of Sex as a Biological Variable in NIH-funded Research.” In that R01 submission, it was only proposed (and subsequently approved) to study female pigs.

*Ex vivo* evaluation of lentiviral transfection

We conducted a series of *ex vivo* pilot studies to evaluate the ability of the lentiviral construct to transfect endothelial cells of pig veins, and the time required for this to occur. We initially used pigs undergoing other procedures and used the freshly harvested femoral vein *ex vivo* to optimize the necessary steps. Because we worked in parallel to develop the AVF model and the procedure to dwell the lentivirus *in vivo* (below), we were able to iteratively develop and optimize a lentiviral article suspension for *in vivo* dwelling, which comprised 8µl polybrene (sc-134220, Santa Cruz), 10µl unfractionated heparin (at 1000 USP units/ml), 500µl nitroglycerin (at 100 µg/ml), 80µl lentiviral particles (at ~5x10^9^ IFU/ml) and 202µl 1x PBS, for a total volume of 800µl. Therefore, for the final *ex vivo* testing of lentiviral transfection, we harvested the untouched femoral vein from 2 separate pigs that had not undergone any prior procedures and after very gentle washing, we pinned the veins out on a moist, flat surface with the intima facing upwards and immediately dwelled the lentiviral article suspension (with heparin, polybrene and nitroglycerin) on the intimal surface of the freshly harvested vein for either 30 or 60 minutes at 37°C. As a control, sections from these same freshly harvested veins were dwelled for 30 minutes at 37°C using the same *ex vivo* techniques with a sham suspension containing all the components described above but without the lentiviral particles. After gentle but repeated washing to completely remove the article, veins were embedded in OCT and frozen at -80°C.

Creation of a preclinical pig AVF model with lentiviral article dwelling

The abdomen and pelvis were prepped with betadine. An incision was made over the right femoral triangle to expose the femoral vein and artery which lie in a groove between the gracilis and sartorius muscles. The fascia overlying the muscles was entered using sharp dissection. The artery and vein were individually dissected free and controlled with silastic vessel loops. Great care was taken in handling the vessels to avoid vasospasm. To allow further mobilization of the vessels, branches of the artery and tributaries of the femoral vein were ligated using 4-0 silk ties. A dose of 5,000 U unfractionated heparin was given intravenously (systemically), prior to clamping the vessels. An atraumatic vascular clamp was then applied to the proximal femoral vein, causing the vein to become distended, which facilitated advancement of a 24 gauge x ¾ inch soft angiocatheter (#SR*FF2419, Terumo, Somerset, NJ) into the vein, 3 cm distal to the vascular clamp. This distance from the vascular clamp to the venipuncture site was standardized, as this segment was designated the treatment area. The angiocatheter was advanced over the needle into the vein until there was blood return. A 1ml syringe was then carefully attached to the catheter and saline was gently flushed into the vein. A second non-traumatic vascular clamp was then applied to the distal aspect of the vein to control inflow. The occluded segment of vein was gently flushed multiple times with saline ensuring that there was no leakage of saline from the vein segment and to flush out residual blood. 800µl of the lentiviral article suspension, as described above, was then injected into the vein via the catheter and allowed to dwell for 60 minutes. The vein remained mildly distended with the article suspension to ensure contact of the article with the endothelium of the vein. After 60 minutes, the clamped vein segment was aspirated and gently flushed with saline to prevent systemic circulation of the article. The angiocatheter was removed from the vein and Potts scissors were used to make a venotomy measuring 1.0 cm. Two 7-0 proline BV-1 sutures were then used as stay sutures to facilitate transposition of the femoral artery to femoral vein. An atraumatic vascular clamp was applied to the proximal end of the femoral artery. The distal end of the artery was ligated with a 4-0 silk tie. The artery was transected and spatulated to the size of the corresponding venotomy. Using 6-0 proline BV-1 suture, an arteriovenous anastomosis was created by transposing the femoral artery to the femoral vein. Prior to completion of the anastomosis, the vascular clamps were removed from the vein and artery to allow back and forward bleeding. After completion of the arteriovenous anastomosis the vein was evaluated for a thrill. The surgical site was irrigated with saline and the skin incision closed with 3-0 vicryl deep dermal sutures and the skin closed with 3-0 monocryl suture in a subcuticular fashion. Animals were then extubated and allowed to recover in a designated recovery room.

While the above steps were followed for Phases 1 and 2 of this study (Figure 1), during the pilot studies in Phase 0 (Supplemental Figure 2) the steps relating to dwelling of the lentiviral article were omitted. The surgical team was A.K. and Y.X. for all surgeries in Phases 0, 1 and 2 of this study. In Phases 1 and 2, the treatment allocation was randomized to either *SMAD3* knockdown or the control group. The primary surgeon for all surgeries in Phase 1 and 2 (A.K. – a practicing clinical vascular surgeon) was fully blinded to the treatment allocation. For logistic reasons, it was necessary that the surgical assistant (Y.X.) was aware of the treatment allocation.

A limited number of surgical complications arose in Phase 2 (but not Phase 1) of this study. In Phase 2, we planned to randomize 8 pigs to receive lentivirus containing *SMAD3* shRNA, and another 8 pigs to receive control lentivirus. However, 4 of the originally randomized pigs (2 per group) were excluded from the study within the first 24 hours after surgery due to procedure-related complications. Three of these pigs were immediately excluded due to surgical complications related to creation of the AVF (2 in the *SMAD3* knockdown group, 1 in the control group), while one pig was excluded the morning after AVF creation due to acute hindlimb ischemia (control group). Therefore, ultimately 10 pigs were randomized into each group, but only 8 pigs per group were evaluated. The 16 pigs (8 per group) that were free of procedure-related complications were housed for 30 days after AVF creation, and comprise the animals presented in the Phase 2 analyses. As examples of comparable human studies and failure rates from the contemporary era, Bassri et al reported an early asymptomatic vein graft failure rate after coronary bypass grafting surgery (CABG) of 10%,^1^ while Zientara reported that early, silent graft failure following off-pump CABG occurred in 33 of 192 asymptomatic patients (17.2%).^2^ Therefore, our rate of technical complications and failure related to the surgical procedure is comparable to the human experience.

**SUPPLEMENTAL FIGURE LEGENDS**

**Supplemental Figure 1. Pictorial representation of steps in the surgical creation of this preclinical AVF model including dwelling of lentiviral article.** **A – K,** The surgical approach to create this preclinical AVF model in pigs was developed by two specialist practicing clinical vascular surgeons in our team (A.K. and R.T.) with input from a specialist large animal surgeon (K.I.), whereby the standard human AVF operation was adapted to the porcine anatomy and the need for dwelling of a lentiviral article. Note that in the initial development and testing of this AVF model during Phase 0, the steps shown in panels E and F (dwelling of lentivirus) were omitted. **L,** The AVF after harvesting, removal of superfluous tissue and prepared for mounting in OCT or paraffin blocks and further analysis.

**Supplemental Figure 2. Pilot study to assess the efficacy of EndMT inhibition in a preclinical large animal AVF model (Phase 0).** **A,** Schematic overview of Phase 0 pilot study. The femoral vein in the untouched left leg (control) and the AVF in the right leg were harvested from 3 female pigs 15 days after the creation of the AVF. No lentivirus or other article was dwelled during AVF creation. **B** and **C,** Representative immunofluorescence staining of EndMT 15 days after AVF creation. Right panels are digital enlargements of the section of the original adjacent images as demarcated by the dashed line. Endothelial markers (CD31 and VE-Cad) are shown in green. Mesenchymal markers (SM22α and αSMA) are in red. DAPI-stained nuclei are in blue. Analyses performed using paired Student’s t test. Scale bar = 50µm. **p* < 0.05. n=3 pigs per group, where each pig was in both the control (untouched left femoral vein) and AVF (right femoral vein) groups.

**Supplemental Figure 3. Assessment of lentiviral construct for *SMAD3* knockdown efficacy in pig endothelial cells. A,** qRT-PCR analysis of the efficacy of *SMAD3* knockdown using a lentiviral construct containing *SMAD3* shRNA versus the same lentiviral construct but containing scramble shRNA control, transfected into pig coronary artery endothelial cells (PCAECs) cultured *in vitro*. Analysis performed using unpaired Student’s t test. n = 3 per group. ***p* < 0.01. **B,** Pilot study showing a section of freshly harvested femoral vein from a pig that had not undergone any prior surgery or treatment, which was freshly harvested and maintained at 37°C *ex vivo* and dwelled either for 30mins with the article suspension that was missing the lentivirus (control), or which was dwelled for 30 or 60 minutes with the article suspension that included the scramble lentivirus. Scale bar = 50µm. **C,** Pilot study with a femoral AVF harvested 24 hours after *in vivo* dwelling for 60 minutes with the article suspension containing the scramble shRNA lentiviral construct. Shown here is a representative image of immunofluorescence staining of the venous limb of the AVF. Scale bar = 50µm. Relevant to B and C, the lentiviral construct expressed GFP, which is seen here with staining for GFP using an anti-GFP primary antibody.

**Supplemental Figure 4. Representative immunofluorescence staining and quantitation of SMAD2 and pSMAD2 in endothelial cells 8 days after AVF creation.** CD31 is shown in green, SMAD2 or pSMAD2 in red, and DAPI in blue. Analyses were performed using unpaired Student’s t test. Scale bars = 50µm. ns, not significant. n=3 pigs per group for both analyses.

**Supplemental Figure 5. Body weights of all 16 pigs in Phase 2 of this study.** Body weights are shown at baseline (immediately after induction of anesthesia prior to lentivirus dwelling and AVF creation) and at the time of terminal harvest (immediately after induction of anesthesia prior to ultrasound, angiography and tissue harvest). For statistical analyses, baseline body weight was compared using Mann-Whitney test and terminal harvest body weight using unpaired Student’s t test.

**Supplemental Figure 6. Femoral angiographic images of all 16 pigs in Phase 2 of this study.** As described in the methods, these images were acquired 30 days after AVF creation, immediately prior to euthanasia and harvesting of the AVF for detailed analysis as shown in Figures 3 – 5.

**Supplemental Figure 7. Ultrasound and angiographic evaluation of the efficacy of EndMT inhibition by *SMAD3* knockdown in preclinical large animal AVF model at 30 days (Phase 2), with exclusion of the 3 AVFs from the control group that were totally occluded.** The analyses shown are the same as in Figure 3, with the only differences being that the 3 AVFs from the control group that were totally occluded have been removed from the analyses, and “reference vein diameter” that is shown in Figure 3C (where the 3 occluded AVFs from the control group have already been excluded) has not been represented. **A**, (corresponds to Figure 3B) Ultrasound measurement at 30 days after AVF creation to assess the surgical anastomotic site and equivalence of AVF creation between groups showing quantifications of the anastomosis diameter, vein area and artery area (as acquired at the anastomosis site). All analyses performed with unpaired Student’s t test. **B**, (corresponds to Figure 3C) Angiographic measurement of AVF diameter, stenosis and patency 30 days after creation. “Minimum vein diameter” represents the minimum diameter of the lentivirus-treated segment of the venous limb of the AVF, “Maximum vein diameter” represents the maximal diameter of the lentivirus-treated segment of the venous limb of the AVF, while “Stenosis of grafted vein” represents the stenosis of the lentivirus-treated segment of the venous limb of the AVF (determined by comparing the minimum with the reference diameters) presented as either % stenosis or the proportion with stenosis <70% versus ≥70%. Minimum vein diameter was compared using Mann-Whitney test. Maximum vein diameter and % stenosis of grafted vein were compared using unpaired Student’s t test. Stenosis of grafted vein (<70% versus ≥70%) was compared using Fisher's exact test. **p* < 0.05; ns, not significant. n=5 pigs in the control group (the 3 pigs with totally occluded AVFs are not included) versus n=8 pigs in the *SMAD3* knockdown group. For analyses that were statistically significant in Figure 3 but which only trend toward significance in this figure, actual *p* values are shown.

**Supplemental Figure 8. Histologic and immunofluorescence evaluation of the efficacy of EndMT inhibition by *SMAD3* knockdown in preclinical large animal AVF model at 30 days (Phase 2), with exclusion of the 3 AVFs from the control group that were totally occluded.** The analyses shown are the same as in Figure 4, with the only difference being that the 3 AVFs from the control group that were totally occluded have been removed from the analyses. **A**, Analyses of inner perimeter, calculated lumen area, and collagen content of the vessel wall based on images using Masson's trichrome staining. Lumen area was calculated from the inner perimeter (i.e. inner circumference) and assuming the vessel was circular in cross-section. For this panel, all images are from the narrowest portion of the venous limb of the AVF. **B**, Quantitation of neointimal thickness (from the intima-media boundary to the intima) for each AVF determined by averaging the neointimal thickness measurement from 3 sites per AVF from a single section using elastic van Gieson (EVG) staining. Images used for this analysis are from close to the narrowest portion of the venous limb of the AVF (within 1 - 2mm). **C**, Quantitation of cells positive for CD31, eNOS and DAPI. **D**, Quantitation of cells positive for VE-Cad, eNOS and DAPI. Images used for quantitation in C and D are from the venous limb of the AVF, within 5 - 10mm of the narrowest portion. Analyses were performed as follows: Panel A - inner perimeter and calculated lumen area with unpaired Student’s t test and collagen content with Mann-Whitney test; Panel B - neointimal thickness with unpaired Student’s t test; Panel C - DAPI^+^ cells with unpaired Student’s t test and other analyses in Panel C with Mann-Whitney test; Panel D - DAPI^+^ cells with unpaired t test and other analyses in Panel D with Mann-Whitney test. **p* < 0.05; ns, not significant. n=5 pigs in the control group (the 3 pigs with totally occluded AVFs are not included) versus n=8 pigs in the *SMAD3* knockdown group. For analyses that were statistically significant in Figure 4 but which only trend toward significance in this figure, actual *p* values are shown.

**Supplemental Figure 9. Evaluation of the effect of EndMT inhibition by *SMAD3* knockdown on cell proliferation, apoptosis and immune cell infiltration in a preclinical large animal AVF model at 30 days (Phase 2), with exclusion of the 3 AVFs from the control group that were totally occluded.** The analyses shown are the same as in Figure 5, with the only difference being that the 3 AVFs from the control group that were totally occluded have been removed from the analyses. Images used for analysis in this Figure are from the venous limb of the AVF, within 5 - 10mm of the narrowest portion. **A**, Quantification of cell proliferation based on immunofluorescence staining for CD31, Ki67 and DAPI. **B**, Quantification of apoptosis based on immunofluorescence staining for TUNEL assay and DAPI. **C**, Quantification of immune cell infiltration based on immunofluorescence staining for CD45 and DAPI. **D**, Quantification of immune cell infiltration based on immunofluorescence staining for CD68 and DAPI. All analyses were performed using Mann-Whitney test except for quantification of cell proliferation in Panel A and CD45+ cell/total DAPI analysis in Panel C, with these two analyses performed with unpaired Student’s t test. ns = not significant. n=5 pigs in the control group (the 3 pigs with totally occluded AVFs are not included) versus n=8 pigs in the *SMAD3* knockdown group.

**SUPPLEMENTAL TABLES**

**Supplemental Table 1.** Primer list for qRT-qPCR.

| **Gene** | **Forward Primer** | **Reverse Primer** |
| --- | --- | --- |
| *SMAD3* | CTGGCTCAGTCTGTCAACCA | CATCTGGGTGAGGACCTTGT |
| *18s* | AATGGGGTTCAACGGGTTAC | TAGAGGGACAAGTGGCGTTC |

**Supplemental Table 2.** List of primary antibodies used for immunostaining.

| **Antibody** | **Company/Source** | **Catalog number** | **Dilution** |
| --- | --- | --- | --- |
| CD31 | Bio-Rad | MCA1746GA | 1:100 |
| SMAD2 | MybioSource | MBS9204752 | 1:100 |
| pSMAD2 | MybioSource | MBS8220276 | 1:100 |
| SMAD3 | Abcam | ab84177 | 1:100 |
| pSMAD3 | Invitrogen | 702292 | 1:100 |
| SM22α | Abcam | ab14106 | 1:100 |
| VE-Cadherin | Cell Signaling Technology | 2500S | 1:100 |
| VE-Cadherin | MybioSource | MBS224684 | 1:100 |
| αSMA | Sigma | A5228 | 1:100 |
| eNOS | Novus Biologicals | NB300-500 | 1:100 |
| Ki67 | Abcam | ab15580 | 1:100 |
| CD45 | Bio-Rad | MAC323 | 1:100 |
| CD68 | Invitrogen | MA5-13324 | 1:100 |
| GFP | Abcam | ab6556 | 1:200 |

**Supplemental Table 3.** List of secondary antibodies used for immunostaining. *Alexa Fluor™ 488 was used exclusively for anti-GFP immunostaining.

| Antibody | Company/Source | Catalog number | Dilution |
| --- | --- | --- | --- |
| Goat anti-rabbit Alexa Fluor™ 488* | Abcam | ab150077 | 1:500 |
| Donkey anti-goat Alexa Fluor™ 546 | Invitrogen | A11056 | 1:500 |
| Donkey anti-rabbit Alexa Fluor™ 546 | Invitrogen | A10040 | 1:500 |
| Goat anti-mouse Alexa Fluor™ 546 | Invitrogen | A21123 | 1:500 |
| Goat anti-rat Alexa Fluor™ 546 | Invitrogen | A11081 | 1:500 |
| Goat anti-mouse Alexa Fluor™ 633 | Invitrogen | A21052 | 1:500 |
| Goat anti-rabbit Alexa Fluor™ 633 | Invitrogen | A21071 | 1:500 |
| Goat anti-rat Alexa Fluor™ 633 | Invitrogen | A21094 | 1:500 |

**SUPPLEMENTAL REFERENCES**

1. Bassri H, Salari F, Noohi F, Motevali M, Abdi S, Givtaj N, Raissi K and Haghjoo M. Evaluation of early coronary graft patency after coronary artery bypass graft surgery using multislice computed tomography angiography. *BMC Cardiovasc Disord*. 2009;9:53.

2. Zientara A, Rings L, Bruijnen H, Dzemali O, Odavic D, Haussler A, Gruszczynski M and Genoni M. Early silent graft failure in off-pump coronary artery bypass grafting: a computed tomography analysisdagger. *Eur J Cardiothorac Surg*. 2019;56:919-925.
